# An LOSM Speed Controller for Autonomous Commercial Vehicles Addressing Disturbance from Load and Slope Uncertainty

**DOI:** 10.3390/s26165203

**Published:** 2026-08-17

**Authors:** Jinwen Yang, Huafu Fang, Ju Lu, Lingang Yang, Zhiqiang Jiang, Giuseppe Carbone

**Affiliations:** 1 School of Electromechanical and Vehicle Engineering, East China Jiaotong University, Nanchang 330013, China; f2751227673@163.com (H.F.); huzai7133@126.com (J.L.); 2024038085500035@ecjtu.edu.cn (L.Y.); giuseppe.carbone@unical.it (G.C.); 2 School of Automotive Engineering, Jiangxi Vocational and Technical College of Communications, Nanchang 330013, China; 3314@ecjtu.edu.cn; 3 Department of Mechanical, Energy, and Management Engineering, University of Calabria, I-87036 Rende, Italy

**Keywords:** autonomous commercial vehicles, Luenberger observer, sliding mode control, velocity tracking, disturbance, uncertainty

## Abstract

Autonomous commercial vehicles (ACVs) frequently encounter drastic variations in payload and complex road conditions during practical operations. Consequently, effectively suppressing external disturbances caused by payload and road slope uncertainties has become a critical challenge in enhancing the robustness of their low-level control systems. To address this issue, this paper proposes a sliding mode control (SMC) strategy based on Luenberger observer disturbance compensation (LOSM), aiming to simultaneously mitigate the adverse effects of these two uncertainties on the vehicle’s speed control performance. First, according to the driving characteristics of commercial vehicles, a full-condition longitudinal dynamic model encompassing uphill, downhill, and flat road scenarios is established. Second, by deeply integrating the Luenberger observer with sliding mode control theory, an active disturbance rejection LOSM speed controller is designed. Furthermore, the boundary conditions for the closed-loop system to achieve asymptotic stability are rigorously derived and proven using Lyapunov functions. Finally, to comprehensively verify the effectiveness of the proposed strategy, eight typical testing scenarios are constructed, and three benchmark algorithms—PI control, radial basis function adaptive sliding mode (RBFSM) control, and radial basis function backstepping sliding mode (RBFBSSM) control are introduced for comparative analysis. The validation results demonstrate that although all four methods can achieve speed tracking and suppress disturbances, the proposed LOSM strategy exhibits the optimal comprehensive performance across various scenarios. Specifically, its steady-state mean error is typically maintained below 2.5%, and it yields the minimum steady-state variance in the majority of scenarios. These results demonstrate that the designed LOSM method can significantly improve the precision and smoothness of ACVs’ speed control under the dual disturbances of unknown mass and road slope.

## 1. Introduction

Driven by factors such as labor shortages caused by an aging population [1] and the surge in traffic pressure triggered by trade globalization [2], the research and development of autonomous commercial vehicles (ACVs) have garnered widespread attention from both academia and industry [3]. This technology holds the promise of significantly reducing driver workload and substantially improving the operational efficiency of traffic flows [4].

As a core module for realizing ACVs, the speed control system coordinates the onboard driving and braking actuators to ensure that the vehicle strictly follows the predefined route, enabling smooth and safe freight transportation in complex road environments. Currently, mainstream speed control methods primarily include proportional-integral-derivative (PID) control [5], fuzzy control [6], linear quadratic regulator (LQR) [7], model predictive control (MPC) [8,9], active disturbance rejection control (ADRC) [10,11], and sliding mode control (SMC) [12,13]. The high precision of traditional PID control relies on explicit system transfer functions for parameter tuning. Due to the strong nonlinear characteristics of commercial vehicle systems, the design of these parameters often lacks rigorous theoretical support. This not only results in extremely high calibration costs but also leads to significant performance degradation in the presence of uncertain external disturbances. To address the challenges of nonlinear systems and the impact of uncertain disturbances, fuzzy control has emerged as an effective alternative to PID control [14,15]. Zhang L et al. [16] proposed a multi-parameter coordinated fuzzy shift control strategy for automated manual transmissions, which significantly improved shifting efficiency compared to conventional methods. Building on this, Ding H et al. [17] introduced a DCT fuzzy shift control strategy based on driving-intention recognition, effectively enhancing ride comfort and driving safety. Nevertheless, the excellent performance of fuzzy control heavily depends on the establishment of a massive rule base and the rational partition of membership functions, requiring extensive engineering experience. Consequently, its design cost is often higher than that of PID control. More importantly, due to the inherent blind spots in expert knowledge, the rationality of fuzzy rules and parameters is difficult to rigorously verify mathematically, which makes the system susceptible to failure risks under extreme operating conditions [18].

To ensure the theoretical rationality and closed-loop stability of control parameters, Lyapunov stability theory provides a rigorous approach. LQR control is based on this theory, seeking the optimal control cost under a quadratic objective function while guaranteeing system stability [19]. Ren Z et al. [20] proposed an improved LQR yaw stability control method considering the influence of road adhesion limits. The results demonstrated that this algorithm could reasonably control the yaw rate and sideslip angle, thereby greatly improving vehicle yaw stability. However, to apply LQR to nonlinear vehicle systems, local linearization is usually required. This approximation inevitably leads to model distortion and a decrease in control precision. Especially under strong uncertain disturbances, the nonlinear characteristics of the system are amplified, further undermining the control effectiveness of LQR [21].

To mitigate the negative impact of model linearization errors on control precision, MPC has become a cutting-edge alternative to LQR [22]. MPC not only eliminates the current tracking error in real-time but also incorporates future prediction errors into consideration through receding horizon optimization. Guo N et al. [23] proposed a real-time predictive control strategy for path following and stabilization of unmanned electric vehicles under extreme driving conditions based on nonlinear model predictive control (NMPC). The validation results showed that this strategy significantly improved steering capability, path-following accuracy, vehicle stability, and real-time performance. However, the improvement in MPC precision is predicated on longer prediction and control horizons, which involve massive online matrix inversions and quadratic programming (QP) calculations. The computational burden at each step is extremely time-consuming, making it difficult to deploy on a large scale within computationally constrained low-level onboard domain controllers [24].

To strike a balance between limited onboard computational power and the demand for strong disturbance rejection, ADRC provides an ideal solution. By designing an extended state observer (ESO), ADRC can estimate the unmodeled external disturbances and the nonlinear dynamics of the system as limped disturbance in real-time, which is then compensated and eliminated by the state error feedback (SEF) control law [25]. Xie H et al. [26] proposed an active disturbance rejection decoupling control strategy, enabling the closed-loop system to achieve the desired control performance even in the presence of uncertainties. Notably, the SEF module in ADRC is often designed in conjunction with an SMC. This is because SMC not only exhibits strong robustness against parameter uncertainties and external disturbances but also allows flexible configuration of the convergence rate of system states. For instance, Zhao J et al. [27] proposed a backstepping sliding mode control strategy based on a radial basis function (RBF) neural network, which effectively suppressed the influence of parameter uncertainties and external disturbances. However, much like the rule design in fuzzy control, the structural design and weight tuning of RBF neural networks are highly empirical. Furthermore, the inherent generalization bottlenecks and inexplicable “black-box” characteristics of neural networks are unfavorable for the engineering mass production deployment of such intelligent SMC algorithms in the low-level execution systems of commercial vehicles, which require stringent functional safety levels.

In light of the aforementioned challenges, this paper proposes an SMC strategy based on Luenberger observer disturbance compensation (LOSM). This strategy not only adapts to the strong nonlinear characteristics of commercial vehicle systems but also effectively compensates for the external disturbances faced by the control system. During actual operations, commercial vehicles frequently experience drastic dynamic changes in freight payload and road slope. Therefore, effectively suppressing the disturbances caused by uncertainties in payload and slope has become a critical and urgent problem in the development of low-level control for ACVs. To this end, the LOSM designed in this paper aims to simultaneously compensate for these two types of uncertain disturbances, thereby comprehensively ensuring the precision and robustness of the speed control for ACVs.

During the design of the control system, this paper first establishes a full-condition longitudinal dynamic model of the vehicle, covering various driving scenarios such as uphill, downhill, and flat roads. Based on this, LOSM speed controllers for driving and braking modes are separately designed according to the dynamic changes in the vehicle’s driving state. By constructing Lyapunov functions, the boundary conditions for the control system to achieve global asymptotic stability are rigorously derived and proven. Simultaneously, to ensure the vehicle’s smoothness during acceleration and deceleration, an adaptive arbitration module for switching between driving and braking states is designed. To verify the effectiveness of the proposed LOSM control strategy, eight typical testing scenarios, including extreme and conventional conditions, are constructed. Three benchmark methods—PI control, radial basis function adaptive sliding mode (RBFSM) control, and radial basis function backstepping sliding mode (RBFBSSM) control are selected for an in-depth comparative analysis.

The remainder of this paper is organized as follows: Section 2 establishes the full-condition longitudinal dynamic model of the vehicle. Section 3 details the design process of the LOSM control strategy, primarily including the tracking controller, braking controller, global asymptotic stability proof, and the construction of the arbitration module. Section 4 provides an in-depth discussion on the comparative validation results across multiple scenarios. Finally, Section 5 concludes the paper.

## 2. Longitudinal Dynamics Modeling Under Full Driving Conditions

### 2.1. Nominal Longitudinal Dynamics Model

**Figure 1:**
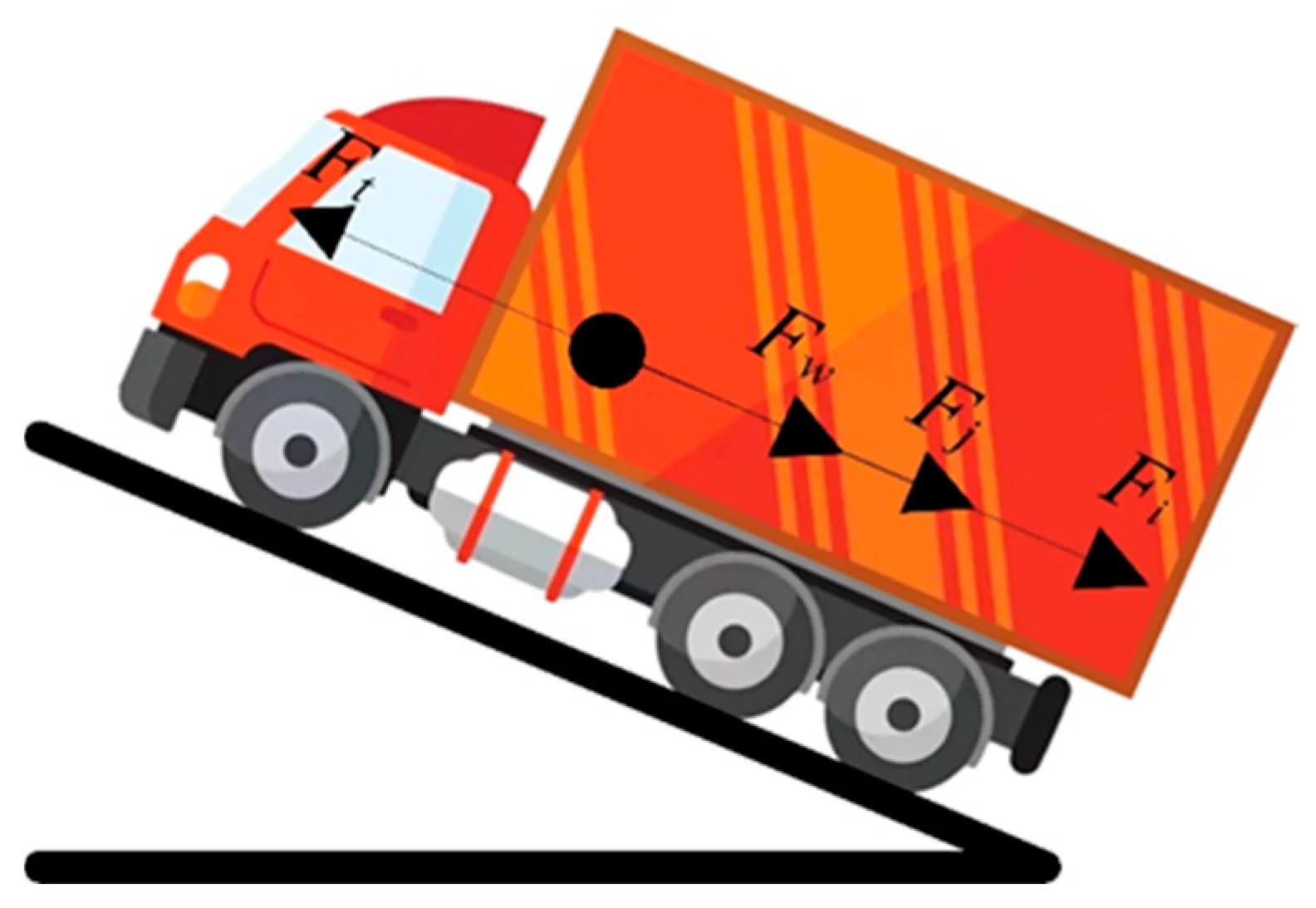
Longitudinal force diagram of vehicle.

As shown in Figure 1, according to Newton’s second theorem, the longitudinal dynamics of an autonomous commercial vehicle can be described by the balance between the controllable longitudinal force and the driving resistances as follows:

(1) $$\left\{\begin{array}{l} F_{x}-F_{f}-F_{w}-F_{j}-F_{i}=m\dot{v}\\ F_{f}=mgf\cos\theta \\ F_{w}=\frac{1}{2}C_{d}A\rho v^{2}\\ F_{j}=\delta_{m}m\frac{dv}{dt}\\ F_{i}=mg\sin\theta \end{array}\right.$$

where *F_x_* is longitudinal control force, *F_f_* is the rolling resistance, *F_w_* is the aerodynamic drag, *F_j_* is the acceleration resistance, *F_i_* is the grade resistance, *m* is the vehicle mass, *v* is the longitudinal vehicle speed, *g* is the gravitational acceleration, *f* is the rolling resistance coefficient, *θ* is the road slope angle, *C_d_* is the aerodynamic drag coefficient, *A* is the frontal area, *ρ* is the air density, *δ_m_* is the is the equivalent rotational mass factor.

To unify the driving and braking modes, the longitudinal control force *F_x_* is defined as

(2) $$F_{x}=\left\{\begin{array}{ll} F_{t} & \mathrm{tracking}\;\mathrm{mode}\\ -F_{b} & \mathrm{braking}\;\mathrm{mode} \end{array}\right.$$

where *F_t_* is the tracking force and *F_b_* is the braking force.

For ease of presentation, Equation (1) is further rewritten as

(3) $$F_{x}-mgf\cos\theta -\frac{1}{2}C_{d}A\rho v^{2}-\delta_{m}m\frac{dv}{dt}-mg\sin\theta =m\dot{v}$$

### 2.2. Longitudinal Dynamics Model Under Different Conditions

In practice, road environments generally include flat, uphill, and downhill conditions. Repetitively deriving a separate dynamic equation for each operating condition leads to redundant expressions and is not conducive to unified controller development. Therefore, Equation (3) is reformulated into the following compact form:

(4) $$F_{x}-\frac{1}{2}C_{d}A\rho V^{2}-d=m\dot{v}$$

where *d* is the lumped disturbance term used to characterize the rolling resistance, acceleration resistance, grade resistance, and their uncertainties, as shown in the following:

(5) $$d=mgf\cos+\delta_{m}m\frac{dv}{dt}+mg\sin\theta$$

When the vehicle travels on a flat road, the grade resistance can be neglected, there is the following:

(6) $$d_{f}=mgf+\delta_{m}m\frac{dv}{dt}$$

where *d_f_* denotes the equivalent disturbance under the flat-road condition.

Thus, the longitudinal dynamics can be expressed as

(7) $$F_{t}-\frac{1}{2}C_{d}A\rho v^{2}-d_{f}=m\dot{v}$$

Under the uphill condition, the grade resistance, the acceleration resistance and rolling resistance impede the forward motion of the vehicle. There is

(8) $$d_{u}=mgf\cos\theta +mg\sin\theta +\delta_{m}m\frac{dv}{dt}$$

where *d_u_* denotes the equivalent disturbance in the uphill condition.

Correspondingly, the vehicle longitudinal dynamic model is given as follows:

(9) $$F_{t}-\frac{1}{2}C_{d}A\rho V^{2}-d_{u}=m\dot{v}$$

When the vehicle travels downhill, the gravity component along the slope assists the vehicle motion. There is

(10) $$d_{d}=mg\sin\theta -mgf\cos\theta -\delta_{m}m\frac{dv}{dt}$$

where *d_d_* denotes the equivalent disturbance in the downhill condition.

To guarantee accurate speed tracking, braking force is usually introduced for speed regulation. In this case, the longitudinal dynamics can be written as follows:

(11) $$-F_{b}-\frac{1}{2}C_{d}A\rho v^{2}+d_{d}=m\dot{v}$$

## 3. LOSM Speed Control Method

To improve the speed-tracking performance of the autonomous commercial vehicle under complex road conditions, including flat, uphill, and downhill scenarios, a mode-dependent longitudinal control framework is adopted. Specifically, a braking control law is designed for the deceleration phase, whereas a driving control law is employed during the acceleration phase. In both modes, a Luenberger disturbance observer is introduced to estimate and compensate for the lumped disturbance online.

### 3.1. Braking Control Law Design

When the vehicle operates under overspeed suppression or downhill speed-limiting conditions, longitudinal deceleration is achieved by regulating the pressure of the braking master cylinder. Let *u*_1_ denote the braking control input. The braking force is defined as

(12) $$F_{b}=k_{1}u_{1},k_{1}>0$$

where *k*_1_ is the braking force coefficient.

Accordingly, the longitudinal dynamics in the braking mode can be written as

(13) $$-k_{1}u_{1}-\frac{1}{2}C_{d}A\rho v^{2}+d_{b}=m\dot{v}$$

where *d_b_* denotes the equivalent disturbance in the braking mode.

The speed-tracking error and sliding surface are defined as

(14) $$s_{b}=v_{d}-v$$

where *v_d_* is the desired driving speed. From Equation (13), there is

(15) $${\dot{s}}_{b}=-\dot{v}=\frac{k_{1}u_{1}}{m}+\frac{C_{d}A\rho v^{2}}{2m}-\frac{d_{b}}{m}$$

To guarantee fast convergence of the system state to the sliding surface, the exponential reaching law is selected as

(16) $${\dot{s}}_{b}=-\varepsilon_{1}\mathrm{sgn}(s_{b})-k_{2}s_{b}$$

where *ε*_1_ > 0 and *k*_2_ > 0 are control parameters.

Combining Equations (15) and (16), the ideal braking control law is obtained as

(17) $$u_{1}^{*}=\frac{d_{b}-\frac{1}{2}C_{d}A\rho v^{2}-m\varepsilon_{1}\mathrm{sgn}(s_{b})-mk_{2}s_{b}}{k_{1}}$$

Since the disturbance *d_b_* cannot be measured directly, the Luenberger disturbance observer is further constructed for online estimation. Define the system state *x_b_* = [*d_b_ v*]^T^, then, the state-space representation in the braking mode is given by

(18) $$\left\{\begin{array}{l} {\dot{x}}_{b}=A_{b}x_{b}+B_{b}u_{1}\\ y_{b}=Cx_{b} \end{array}\right.$$

where $A_{b}=\left[\begin{array}{cc} 0 & 0\\ \frac{1}{m} & -\frac{C_{d}A\rho v}{2m} \end{array}\right]$, $B_{b}=\left[\begin{array}{c} 0\\ -\frac{k_{1}}{m} \end{array}\right]$, $C=\left[\begin{array}{cc} 0 & 1 \end{array}\right]$.

It should be noted that the mass used in the controller and observer design refers to the nominal mass, which is treated as a fixed design parameter. In the uncertain-load scenarios, the actual vehicle mass is allowed to deviate from this nominal value. The primary effect of the resulting mass mismatch is reflected in the lumped disturbance *d_b_*, which is estimated online by the Luenberger observer and compensated through the feedforward term in the control law. Therefore, the proposed framework does not require the exact value of the actual mass, provided that the observer dynamics are sufficiently fast to track the combined effects of mass variation and other disturbances.

Accordingly, the observer is designed as

(19) $$\left\{\begin{array}{l} {\dot{\hat{x}}}_{b}=A_{b}{\hat{x}}_{b}+B_{b}u_{1}+L_{b}\left(y_{b}-{\hat{y}}_{b}\right)\\ {\hat{y}}_{b}=C{\hat{x}}_{b} \end{array}\right.$$

where *L_b_* = [*l_b_*_1_ *l_b_*_2_]^T^ is the observer gain vector. It should be noted that the observer design in this section is conducted under individual constant-speed operating conditions. For each specific scenario, the longitudinal vehicle speed *v* is treated as a fixed parameter, and consequently the system matrix *A_b_* in Equation (18) becomes a constant matrix. This allows the direct application of the pole-placement method for computing the observer gain *L_b_*. The validity of the proposed observer is then evaluated across multiple constant-speed cases in the simulation section.

Define the observation error as follows:

(20) $$e_{b}=x_{b}-{\hat{x}}_{b}$$

According to Equations (15) and (16), there is

(21) $${\dot{e}}_{b}=(A_{b}-L_{b}C)e_{b}$$

The corresponding characteristic equation is

(22) $$\lambda^{2}+\left(\frac{C_{d}A\rho v}{2m}-l_{b2}\right)\lambda +\frac{l_{b1}}{m}=0$$

By pole placement, if the observer double poles are assigned at −*p_b_*, the observer gains can be obtained as

(23) $$\left\{\begin{array}{l} l_{b1}=mp_{b}^{2}\\ l_{b2}=2p_{b}-\frac{C_{d}A\rho v}{2m} \end{array}\right.$$

Replacing the unknown disturbance *d_b_* with its estimate d^b, the implementable braking control law is obtained as

(24) $$u_{1}=\frac{{\hat{d}}_{b}-\frac{1}{2}C_{d}A\rho v^{2}-m\varepsilon_{1}\mathrm{sgn}(s_{b})-mk_{2}s_{b}}{k_{1}}$$

Remark on chattering suppression. Although the proposed sliding mode control law adopts the discontinuous sign function sgn(*s_b_*), which may induce high-frequency chattering in conventional SMC, the chattering effect is mitigated to some extent in the proposed LOSM framework through the Luenberger observer. Specifically, the observer provides a smooth continuous estimate of the lumped disturbance, which is then fed forward into the sliding mode control law. This observer-based feedforward structure introduces a low-pass filtering effect on the control signal, which helps to reduce high-frequency components that typically cause chattering.

To analyze the closed-loop stability, the Lyapunov function is selected as

(25) $$V_{b}=\frac{1}{2}{s_{b}}^{2}$$

Then there is

(26) $${\dot{V}}_{b}=s_{b}{\dot{s}}_{b}=s_{b}(-\varepsilon_{1}\mathrm{sgn}(s_{b})-k_{2}s_{b})=-\varepsilon_{1}\left|s_{b}\right|-k_{2}{s_{b}}^{2}-\frac{s_{b}}{m}{\tilde{d}}_{b}$$

where d˜b=db−d^b denotes the disturbance estimation error under braking condition. When the observer converges such that ${\overset{˜}{d}}_{b}\to 0$, there is

(27) $${\dot{V}}_{b}=-\varepsilon_{1}\left|s_{b}\right|-k_{2}{s_{b}}^{2}\leq 0$$

Therefore, the braking closed-loop system is asymptotically stable.

### 3.2. Tracking Control Law Design

Under starting, acceleration, or uphill traction operating conditions, the driving actuator is utilized to generate the driving force for longitudinal speed tracking control. Defining the tracking control input as *u*_2_ the driving force is given by the following:

(28) $$F_{t}=k_{3}u_{2},k_{3}>0$$

where *k*_3_ is the tracking force coefficient.

Accordingly, the longitudinal dynamics in the tracking mode can be written as

(29) $$k_{3}u_{2}-\frac{1}{2}C_{d}A\rho v^{2}-d_{t}=m\dot{v}$$

where *d_t_* denotes the equivalent disturbance in the tracking mode.

Similarly, the speed-tracking error and sliding surface are defined as

(30) $$s_{t}=v_{d}-v$$

From Equation (29), there is

(31) $${\dot{s}}_{t}=-\dot{v}=-\frac{k_{3}u_{2}}{m}+\frac{C_{d}A\rho v^{2}}{2m}+\frac{d_{t}}{m}$$

Similarly, the exponential reaching law is selected as

(32) $${\dot{s}}_{t}=-\varepsilon_{2}\mathrm{sgn}(s_{t})-k_{4}s_{t}$$

where *ε*_2_ > 0 and *k*_4_ > 0 are control parameters.

Combining Equations (31) and (32), the ideal tracking control law is obtained as

(33) $$u_{2}^{*}=\frac{d_{t}+\frac{1}{2}C_{d}A\rho v^{2}+m\varepsilon_{2}\mathrm{sgn}(s_{t})+mk_{4}s_{t}}{k_{3}}$$

To achieve online estimation of the unknown disturbance *d_t_*, a Luenberger disturbance observer is also constructed. Define the system state *x_t_* = [*d_t_ v*]^T^, then, the state-space representation in the tracking mode is given by the following:

(34) $$\left\{\begin{array}{l} {\dot{x}}_{t}=A_{t}x_{t}+B_{t}u_{2}\\ y_{t}=Cx_{t} \end{array}\right.$$

where $A_{t}=\left[\begin{array}{cc} 0 & 0\\ -\frac{1}{m} & -\frac{C_{d}A\rho v}{2m} \end{array}\right]$, $B_{t}=\left[\begin{array}{c} 0\\ \frac{k_{3}}{m} \end{array}\right]$.

Accordingly, the observer is designed as

(35) $$\left\{\begin{array}{l} {\dot{\hat{x}}}_{t}=A_{t}{\hat{x}}_{t}+B_{t}u_{2}+L_{t}\left(y_{t}-{\hat{y}}_{t}\right)\\ {\hat{y}}_{t}=C{\hat{x}}_{t} \end{array}\right.$$

where *L_t_* = [*l_t_*_1_ *l_t_*_2_]^T^ is the observer gain vector.

Define the observation error as

(36) $$e_{t}=x_{t}-{\hat{x}}_{t}$$

According to Equations (34) and (35), there is

(37) $${\dot{e}}_{t}=(A_{t}-L_{t}C)e_{t}$$

The corresponding characteristic equation is

(38) $$\lambda^{2}+\left(\frac{C_{d}A\rho v}{2m}+l_{t2}\right)\lambda -\frac{l_{t1}}{m}=0$$

By pole placement, if the observer double poles are assigned at −*p_t_*, the observer gains can be obtained as

(39) $$\left\{\begin{array}{l} l_{t1}=-mp_{t}^{2}\\ l_{t2}=2p_{t}-\frac{C_{d}A\rho v}{2m} \end{array}\right.$$

Therefore, the implementable tracking control law can be expressed as

(40) $$u_{2}=\frac{{\hat{d}}_{t}+\frac{1}{2}C_{d}A\rho v^{2}+m\varepsilon_{2}\mathrm{sgn}(s_{t})+mk_{4}s_{t}}{k_{3}}$$

Select the Lyapunov function as

(41) $$V_{t}=\frac{1}{2}{s_{t}}^{2}$$

Then, there is

(42) $${\dot{V}}_{t}=s_{t}{\dot{s}}_{t}=-\varepsilon_{2}\left|s_{t}\right|-k_{4}{s_{t}}^{2}+\frac{s_{t}}{m}{\tilde{d}}_{t}$$

where d˜t=dt−d^t denotes the disturbance estimation error under tracking condition.

When the observer converges such that ${\overset{˜}{d}}_{t}\to 0$, there is

(43) $${\dot{V}}_{t}=-\varepsilon_{2}\left|s_{t}\right|-k_{4}{s_{t}}^{2}\leq 0$$

Therefore, the tracking closed-loop system is asymptotically stable.

### 3.3. Design of Tracking and Braking Arbitration Strategy

Since the tracking system and the braking system do not operate simultaneously during vehicle operation, the longitudinal control arbitration rule is designed based on the vehicle speed error as follows:

(44) $$\left\{\begin{array}{ll} F_{t}=k_{3}u_{2},F_{b}=0 & \;v_{err}>0\\ F_{t}=0,F_{b}=0 & \;v_{t}\leq v_{err}\leq 0\\ F_{t}=0,F_{b}=k_{1}u_{1} & \;v_{err}<v_{t} \end{array}\right.$$

where *v_err_* denotes the vehicle speed error, and *v_t_* represents the speed switching threshold.

**Figure 2:**
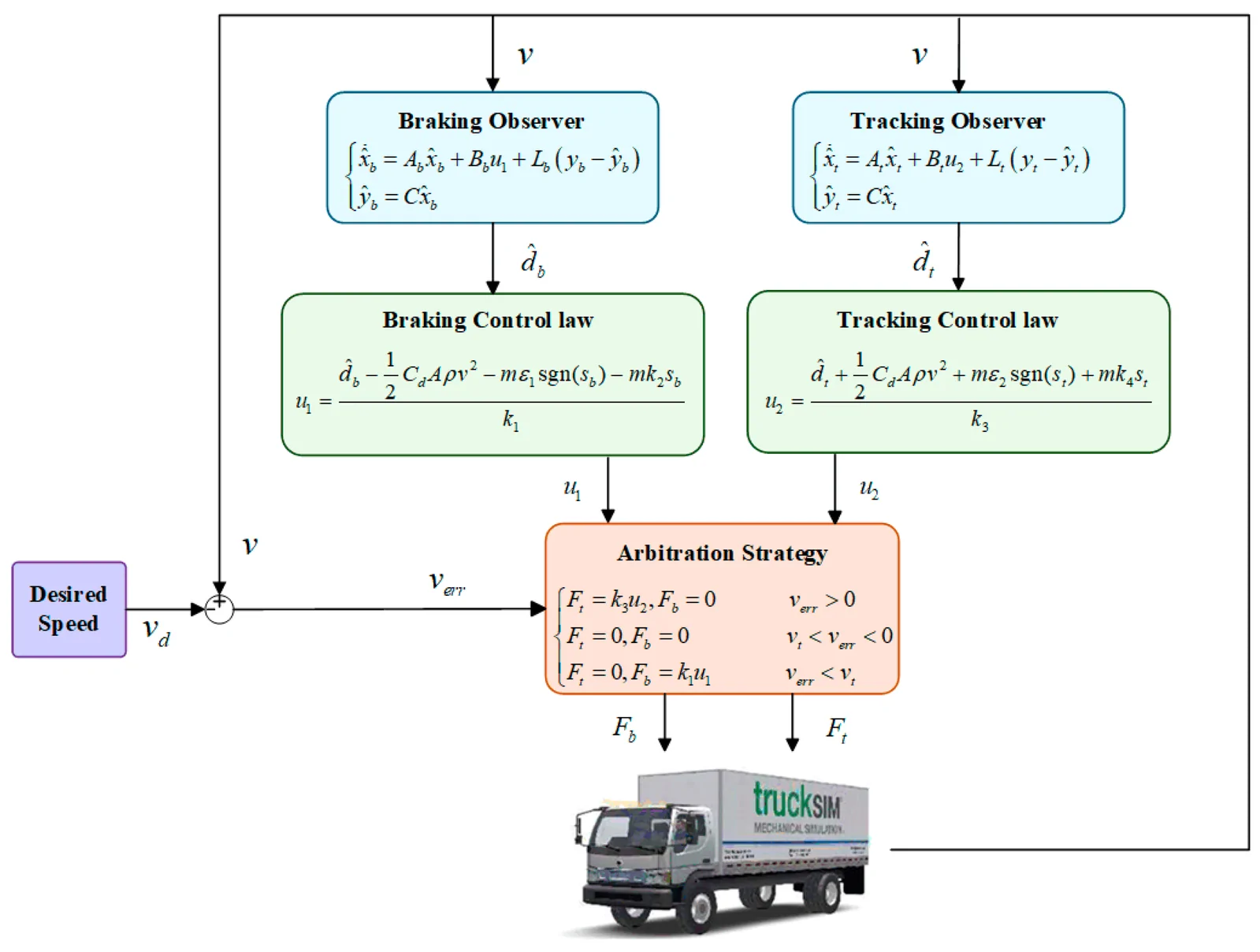
Vehicle control block diagram.

In summary, the block diagram of the vehicle longitudinal speed controller proposed in this paper is shown in Figure 2.

It should be noted that in the above switching logic, the observer adopts the same structure across the traction, coasting, and braking modes, with only the parameters in the system matrix *A* and control matrix *B* differing. Since the observer states are the vehicle speed *v* and the disturbance estimate, and the vehicle speed is physically continuous at the switching instants while the disturbance estimate as an integral output of the observer does not jump, the mode-switching process is smooth. Meanwhile, the piecewise switching conditions based on different ranges of speed error ensure the continuity of the control force at the switching boundaries. The simulation results show smooth speed responses during uphill-to-flat and flat-to-downhill transitions as well as near mode-switching points in the comprehensive road scenarios, validating the effectiveness of the switching strategy.

### 3.4. Coupled Observer–Controller Stability Analysis

The Lyapunov analyses in Section 3.1 and Section 3.2 are conducted under the assumption that the observer error has converged (i.e., ${\overset{˜}{d}}_{b}\to 0$ and ${\overset{˜}{d}}_{t}\to 0$). This section further analyzes the system stability when the observer error has not fully converged. The derivation is presented for the braking mode.

According to Equation (21), through pole placement, the observer poles are assigned at −*p_b_*, where *p_b_* > 0, so (*A_b_* − *L_b_C*) is Hurwitz. There exist positive constants *γ* and *λ*_0_ such that

(45) $$\left\|e_{b}(t)\right\|\leq \gamma e^{-\lambda_{0}t}\left\|e_{b}(0)\right\|$$

That is, the observer error subsystem is globally exponentially stable.

From Equations (15) and (16), substituting Equation (24) control law into the sliding surface dynamics yields

(46) $${\dot{s}}_{b}=-\varepsilon_{1}\mathrm{sgn}(s_{b})-k_{2}s_{b}+\frac{1}{m}{\tilde{d}}_{b}$$

When ${\overset{˜}{d}}_{b}=0$, the system reduces to the nominal case analyzed in Section 3.1, which is asymptotically stable by the Lyapunov function $V=\frac{1}{2}S_{b}^{2}$. When ${\overset{˜}{d}}_{b}\neq 0$, ${\overset{˜}{d}}_{b}$ acts as an external input to the sliding surface dynamics. Since ${\overset{˜}{d}}_{b}$ decays exponentially to zero (as guaranteed by Equation (45)) and the nominal system is asymptotically stable, the closed-loop system is asymptotically stable for each individual constant-speed operating condition according to cascaded systems stability theory. The coupled stability analysis for the tracking mode follows the exact same form and is therefore omitted for brevity.

It should be noted that a fixed set of observer gains is used across all simulation scenarios in this work, which is designed via pole placement at a specific constant speed. The above stability analysis is based on the premise that the vehicle speed remains constant within each simulation scenario.

## 4. Examples Illustration

### 4.1. Simulation Environment Setup

In this paper, Matlab/Simulink 2018b and Trucksim 2019 were used to jointly simulate to verify the effectiveness of the control algorithm. Trucksim selected the vehicle model and set the road condition, while Simulink built the controller. The air resistance coefficient is 1.206, the windward area of the vehicle is 6.8 m^2^ and the air density is 1.32 kg∙m^−3^. The key tuning parameters of the proposed LOSM controller are as follows: the observer poles for both braking and tracking modes are placed at −*p_b_* = −*p_t_* = −2, and the sliding mode control parameters are set to *ε*_1_ = *ε*_2_ = 0.001, *k*_1_ = 0.00033, *k*_2_ = 20, *k*_3_ = 0.25, *k*_4_ = 80.

To verify the effectiveness of the proposed speed control method, eight typical scenarios are set up, and three types of comparison algorithms are introduced to carry out comparison verification.

Considering the interference of load and gradient uncertainty, eight typical testing scenarios are designed as listed below:

(1) Load certain and level road driving;
(2) Load uncertain and level road driving;
(3) Load certain and gradient uncertain in uphill road driving;
(4) Load uncertain and gradient uncertain in uphill road driving;
(5) Load certain and gradient uncertain in downhill road driving;
(6) Load uncertain and gradient uncertain in downhill road driving;
(7) Load certain and slope uncertain in comprehensive road driving;
(8) Load uncertain and slope uncertain in comprehensive road driving.

To verify the effectiveness of the proposed vehicle controller for speed tracking on a level road with constant mass, three strategies, namely, PI, RBFSM, and RBFBSSM, are used to compare the tracking effects.

### 4.2. Results of Eight Scenarios

#### 4.2.1. Scenario 1: Load Certain and Level Road Driving

**Figure 3:**
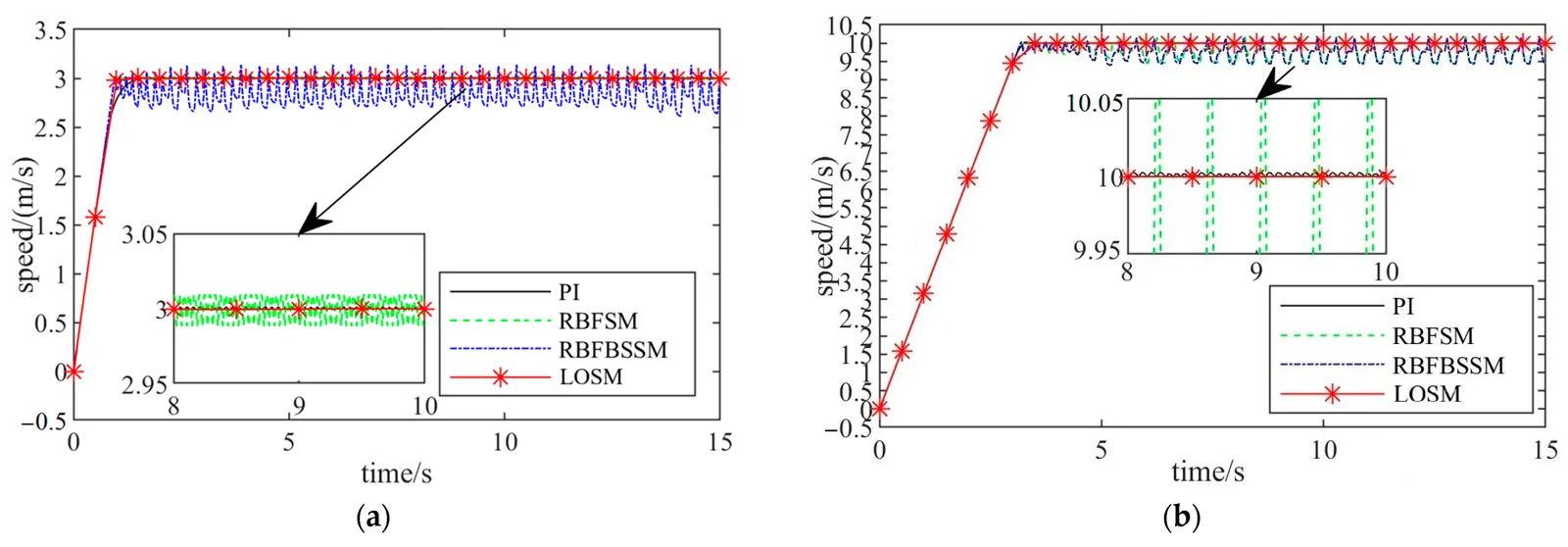
The control performance of certain load on level roads: (**a**) 3 m/s; (**b**) 10 m/s.

**Figure 4:**
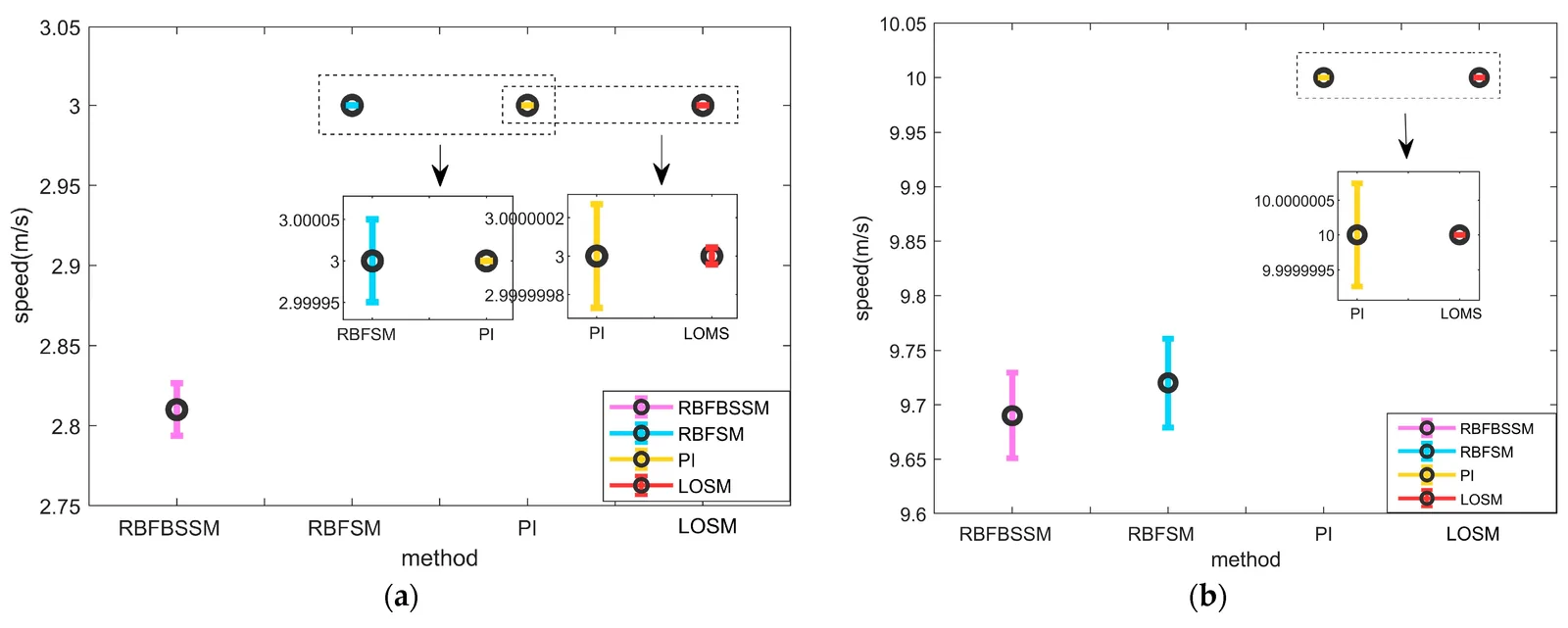
The speed error bars of certain load on level roads: (**a**) 3 m/s; (**b**) 10 m/s.

The parameters are set as follows: the vehicle mass is 3000 kg, the road slope is 0°. The speed is set as 3 m/s and 10 m/s, respectively. To avoid the combinatorial explosion of test cases, only the vehicle speed is varied in scenario 1, while in the remaining scenarios the speed is fixed at 3 m/s and the primary variations are introduced through load condition, road gradient, or their combination. The control performances of certain load on level road are shown in Figure 3 and Figure 4 and Table 1.

As can be seen from Figure 3, the four methods all show higher control accuracy, but the LOSM algorithm has faster response speed and lower frequency of buffering than the other three algorithms. In addition, the speed curve of the proposed LOSM method is visually smoother than those of the benchmark algorithms, with no discernible high-frequency oscillations. This qualitative observation confirms that the observer-based feedforward structure effectively mitigates chattering, ensuring that the superior accuracy of LOSM is not accompanied by undesirable oscillations. As shown in Table 1, the steady-state mean error of RBFBSSM is relatively large. As can be seen from Figure 4, the LOSM algorithm not only maintains the minimum steady-state mean value error, but also has the smallest steady-state variance among the four methods. In other words, the LOSM algorithm is better than the other three algorithms in tracking target speed on level road.

#### 4.2.2. Scenario 2: Load Uncertain and Level Road Driving

**Figure 5:**
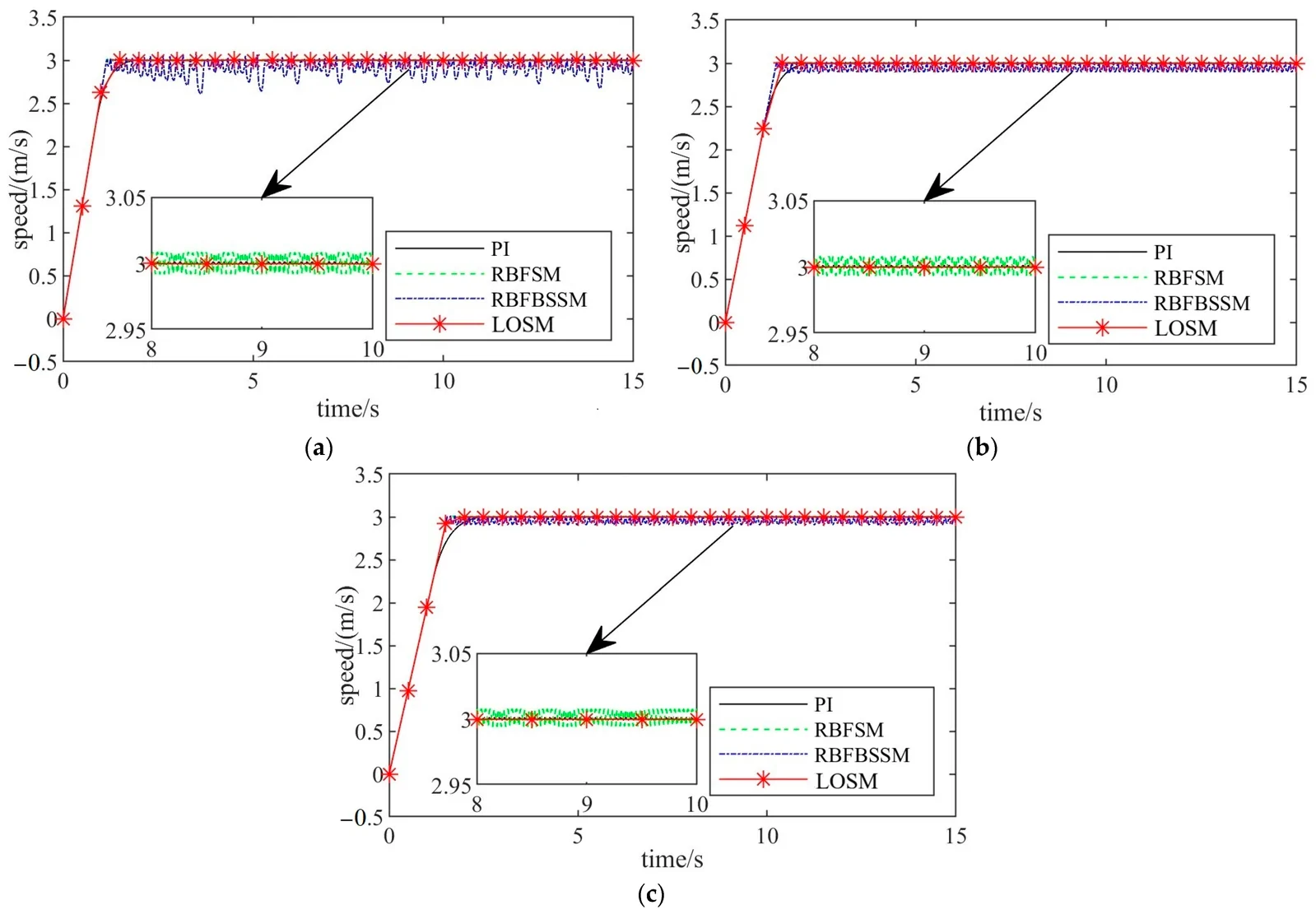
The control performance of uncertain load on level roads: (**a**) 4000 kg; (**b**) 5000 kg; (**c**) 6000 kg.

**Figure 6:**
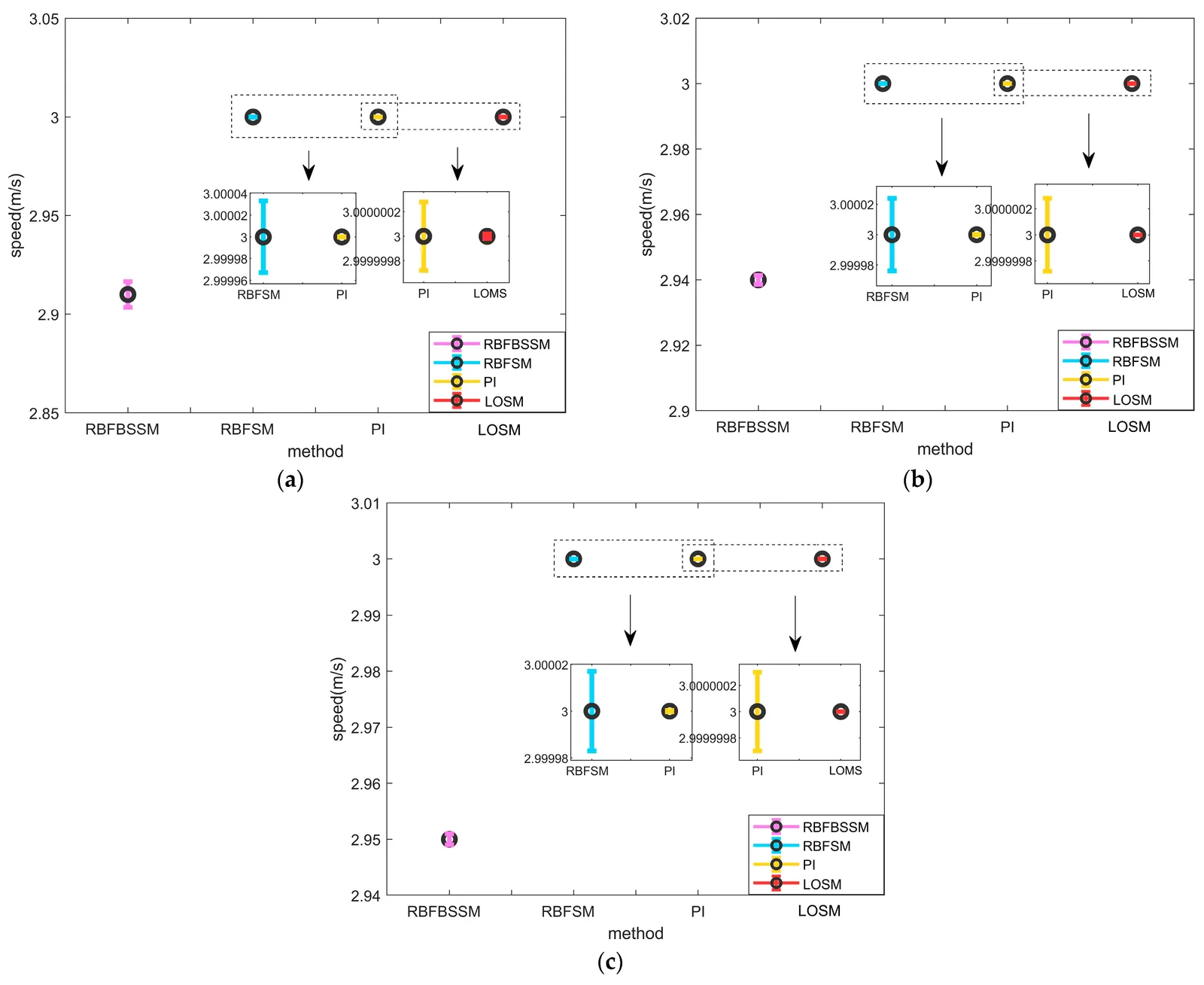
The speed error bars of uncertain load on level roads: (**a**) 4000 kg; (**b**) 5000 kg; (**c**) 6000 kg.

During the use of automatic commercial vehicles, from starting point to destination, the load quality is not always fixed and is generally no-load, half-load and full load, etc. In order to verify the anti-interference ability of the LOSM algorithm in the face of load uncertainty on level roads, the road slope is set to 0°. The load uncertainty Δ*m* is set as 1000 kg, 2000 kg and 3000 kg, respectively, and the target speed is set as 3 m/s. The control performances of uncertain load on level roads are shown in Figure 5 and Figure 6 and Table 2.

The results in Table 2 show that the RBFBSSM algorithm has a large error when tracking the target speed with different load mass, and the maximum steady-state mean error can reach 0.09 m/s, while the other three methods all show high control accuracy. Moreover, as shown in Figure 5a–c, the LOSM algorithm not only has the smallest steady-state mean error, but also the smallest steady-state variance among algorithms of different qualities, which further indicates that the LOSM algorithm has the ability to overcome the interference of load uncertainty when driving on roads with defined slope.

#### 4.2.3. Scenario 3: Load Certain and Gradient Uncertain in Uphill Road Driving

**Figure 7:**
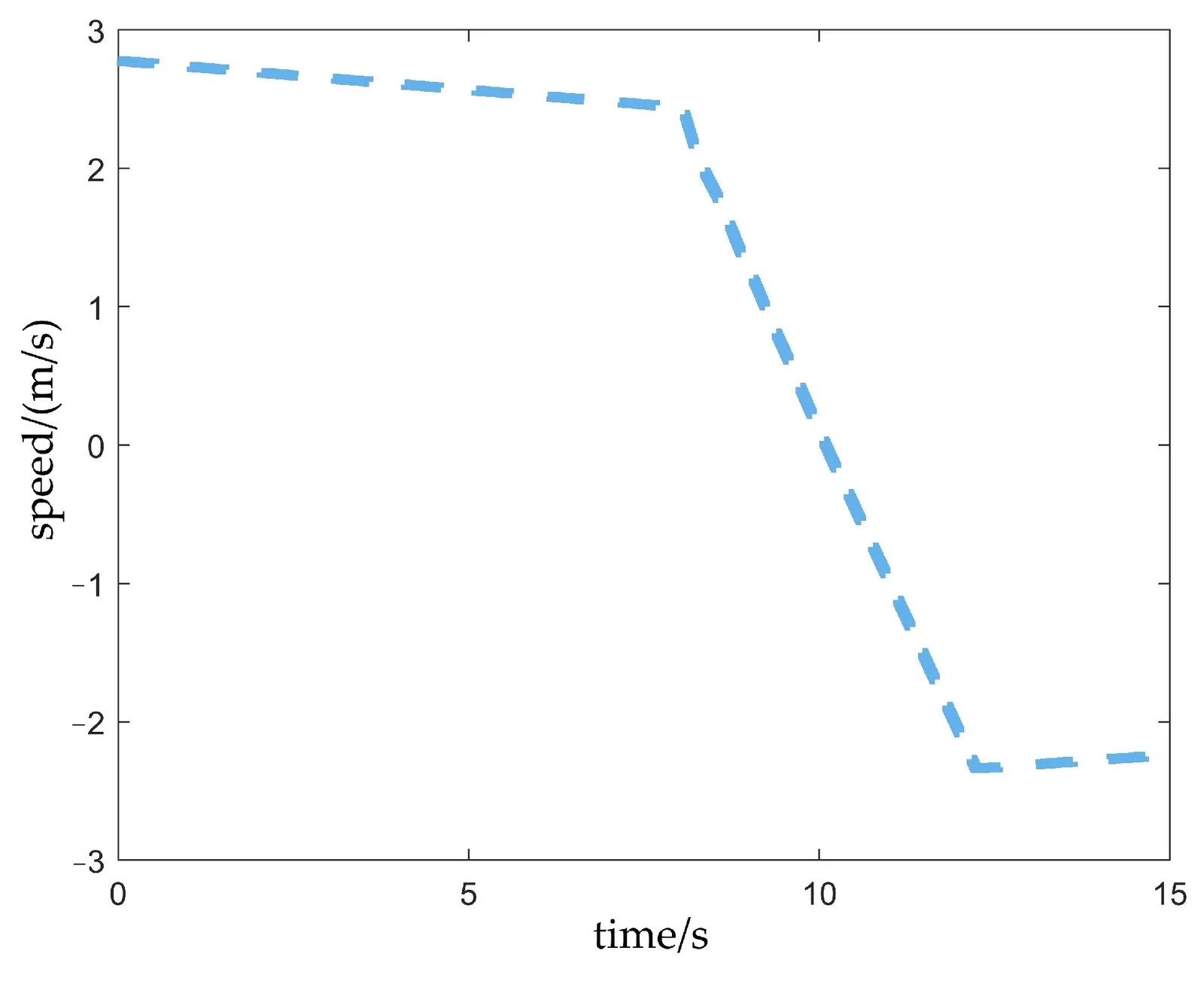
Uphill speed curve without control.

When a conventional commercial vehicle goes uphill, the driver needs to control the throttle pressure properly to keep the vehicle climbing at a steady speed. For ACVs, the driver is no longer in the loop. As the vehicle travels from flat terrain onto an uphill slope with an initial speed, its velocity will decay to zero and eventually become negative without supplementary control, as shown in Figure 7. Such rollback behavior is highly hazardous. Thus, torque control is necessary to track the target speed and guarantee stability during gradient ascent.

**Figure 8:**
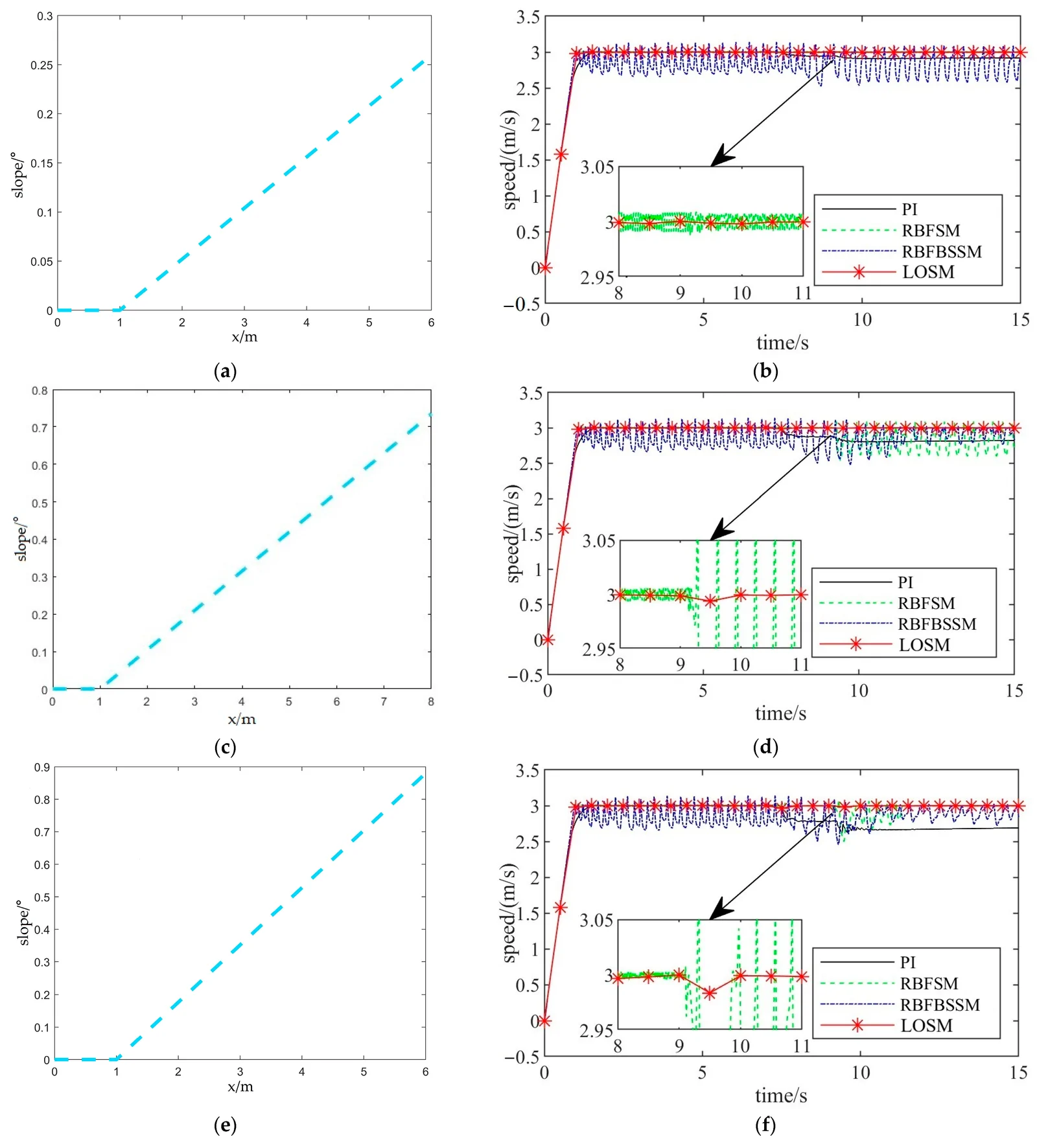
The control performance of certain load and gradient uncertain on uphill roads: (**a**) 3° slope; (**b**) speed under 3° slope; (**c**) 6° slope; (**d**) speed under 6° slope; (**e**) 10° slope; (**f**) speed under 10° slope.

**Figure 9:**
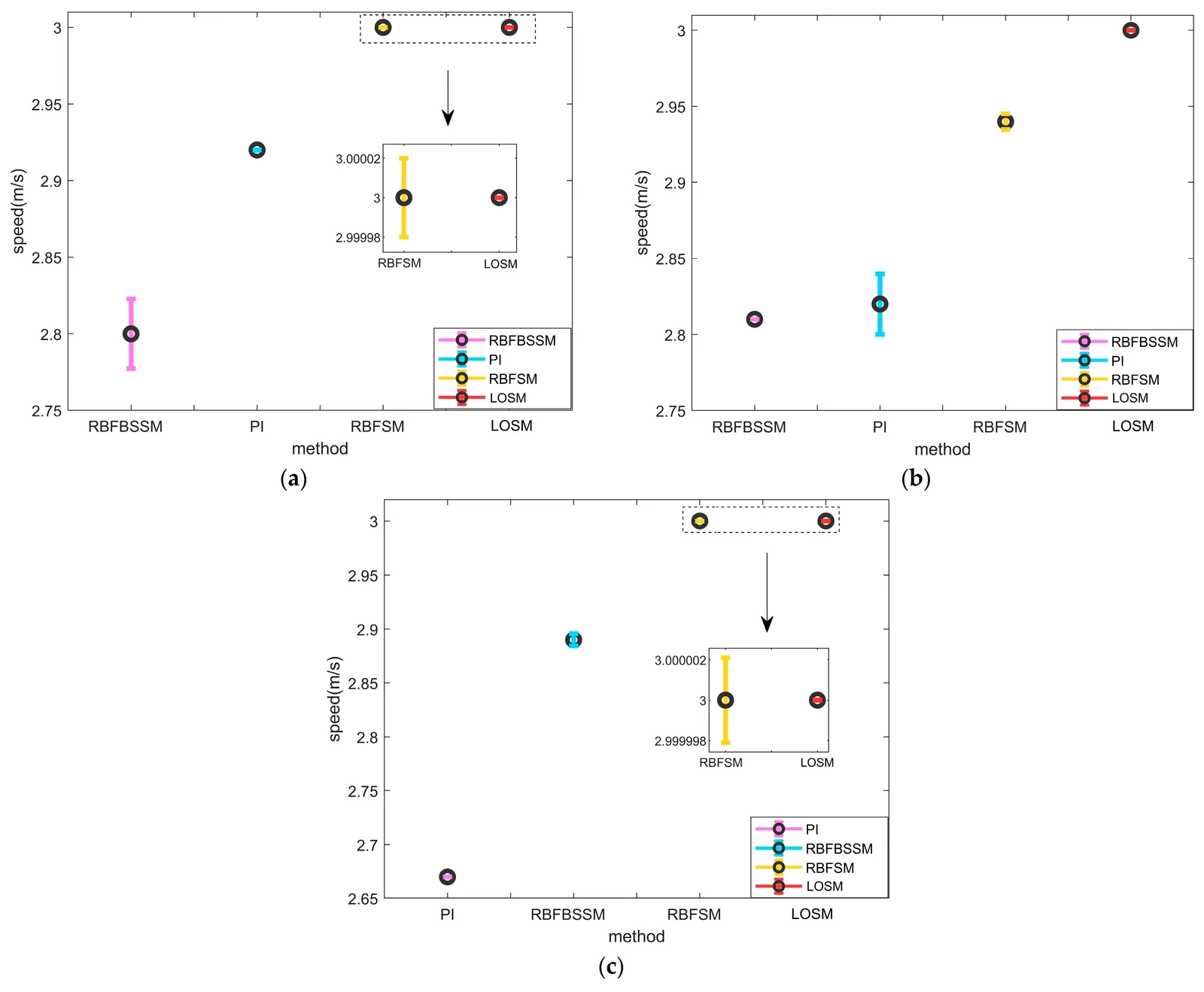
The speed error bars of certain load and gradient uncertain on uphill roads: (**a**) 3°; (**b**) 6°; (**c**) 10°.

In order to verify the effectiveness of the LOSM in the face of unknown uphill slope interference when determining the vehicle load, the load is set as 3000 kg; the slope uncertainty Δ*a* is set to 3°, 6° and 10°, respectively, and the target speed is set to 3 m/s. The control performances are shown in Figure 8 and Figure 9 and Table 3.

As shown in Figure 8a,c,e, road slope is introduced from the longitudinal position of 1 m as an uncertain disturbance of the control system. As shown in Figure 8b,d,f, under the influence of slope uncertainty interference, the PI controller has a significant increase in steady-state error, and the LOSM is relatively small. As shown in Table 4, the mean steady-state errors of the PI and RBFBSSM increase gradually with increasing slope gradient, while the error of the LOSM stays nearly at zero. As shown in Figure 9, compared with the above three algorithms, the LOSM algorithm proposed in this paper can stably track the target speed in the face of the influence of slope uncertainty interference, and the error bar is the smallest among the algorithms compared with the three different controllers. The results show that the LOSM algorithm has better robustness and stability in the face of slope uncertainty interference than the other three methods.

#### 4.2.4. Scenario 4: Load Uncertain and Gradient Uncertain in Uphill Road Driving

**Figure 10:**
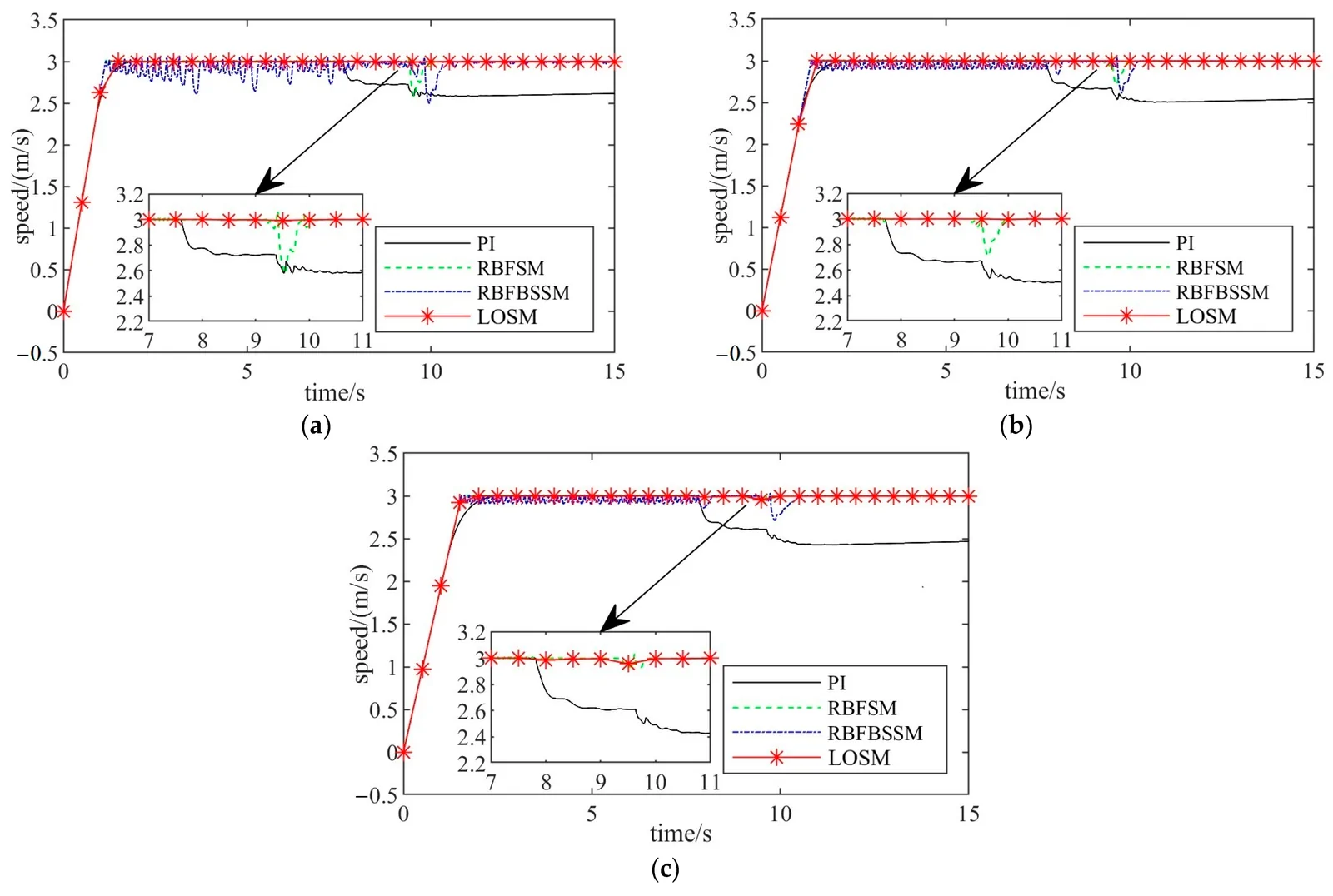
The control performance of uncertain load on gradient on uphill roads: (**a**) 4000 kg; (**b**) 5000 kg; (**c**) 6000 kg.

**Figure 11:**
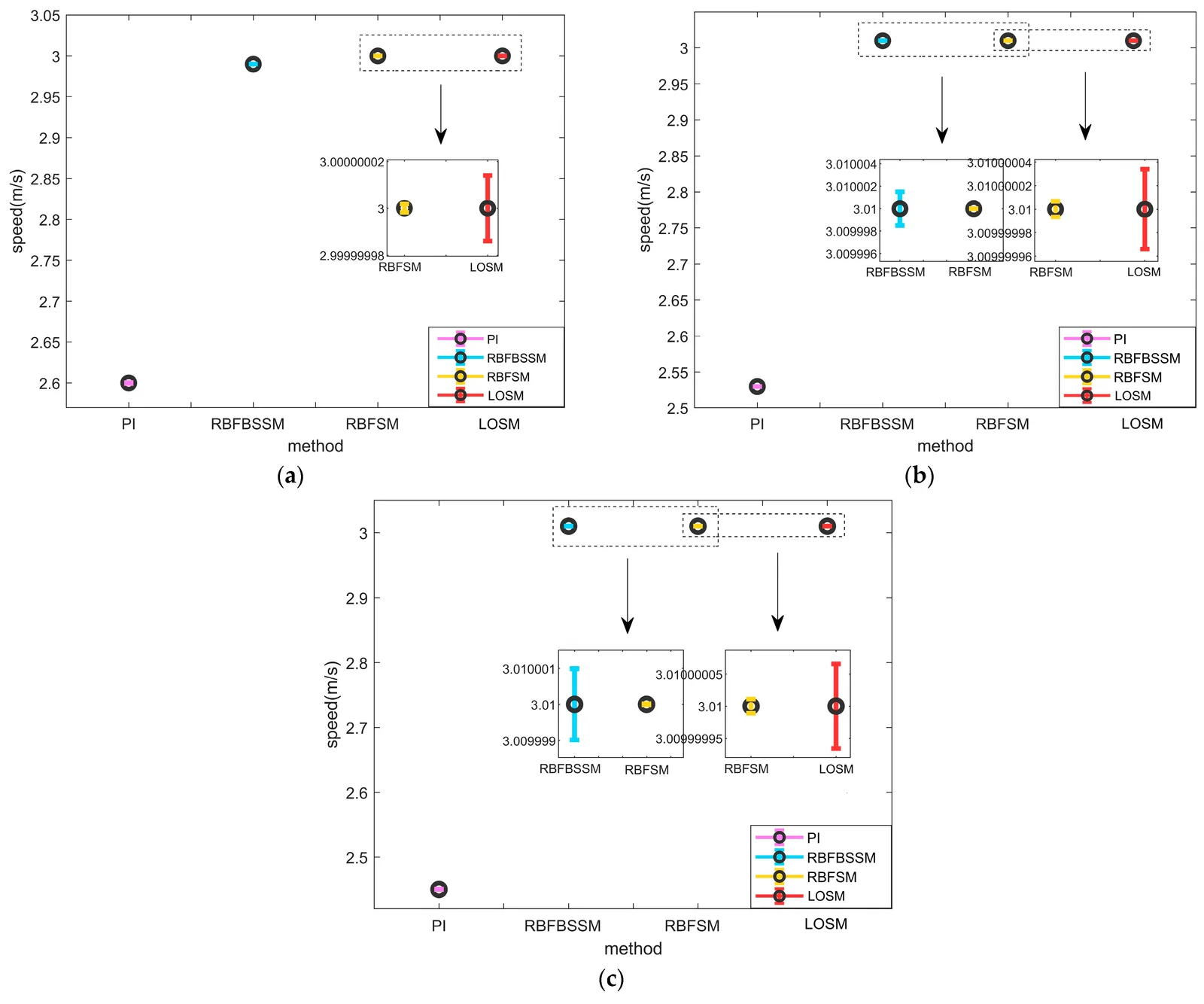
The error bar of uncertain load on gradient on uphill roads: (**a**) 4000 kg; (**b**) 5000 kg; (**c**) 6000 kg.

Scenario 3 only compares vehicles facing different slope disturbances. In order to verify the effectiveness of LOSM in speed tracking when facing dual disturbances of load change and slope change on uphill roads, the vehicle mass is set to 3000 kg, 4000 kg, 5000 kg and 6000 kg, respectively, the slope is set to 10°, and the target speed is set at 3 m/s. The control performances are shown in Figure 10 and Figure 11 and Table 4.

As can be seen from Figure 10, compared with other control strategies, the LOSM controller exhibits the smallest vehicle speed fluctuation when counteracting the dual disturbances arising from coupled gradient and load variations. Table 4 shows that the LOSM algorithm achieves good tracking of the target speed with smallest steady-state errors. As can be seen from Figure 11, despite being slightly less stable than the RBFBSSMC, the LOSM algorithm consistently maintains robust tracking performance under unknown slope gradients and unknown payload disturbances across all tested conditions. In summary, the LOSM algorithm demonstrates superior overall performance over the other three algorithms in uphill target speed tracking tasks.

#### 4.2.5. Scenario 5: Load Certain and Gradient Uncertain in Downhill Road Driving

**Figure 12:**
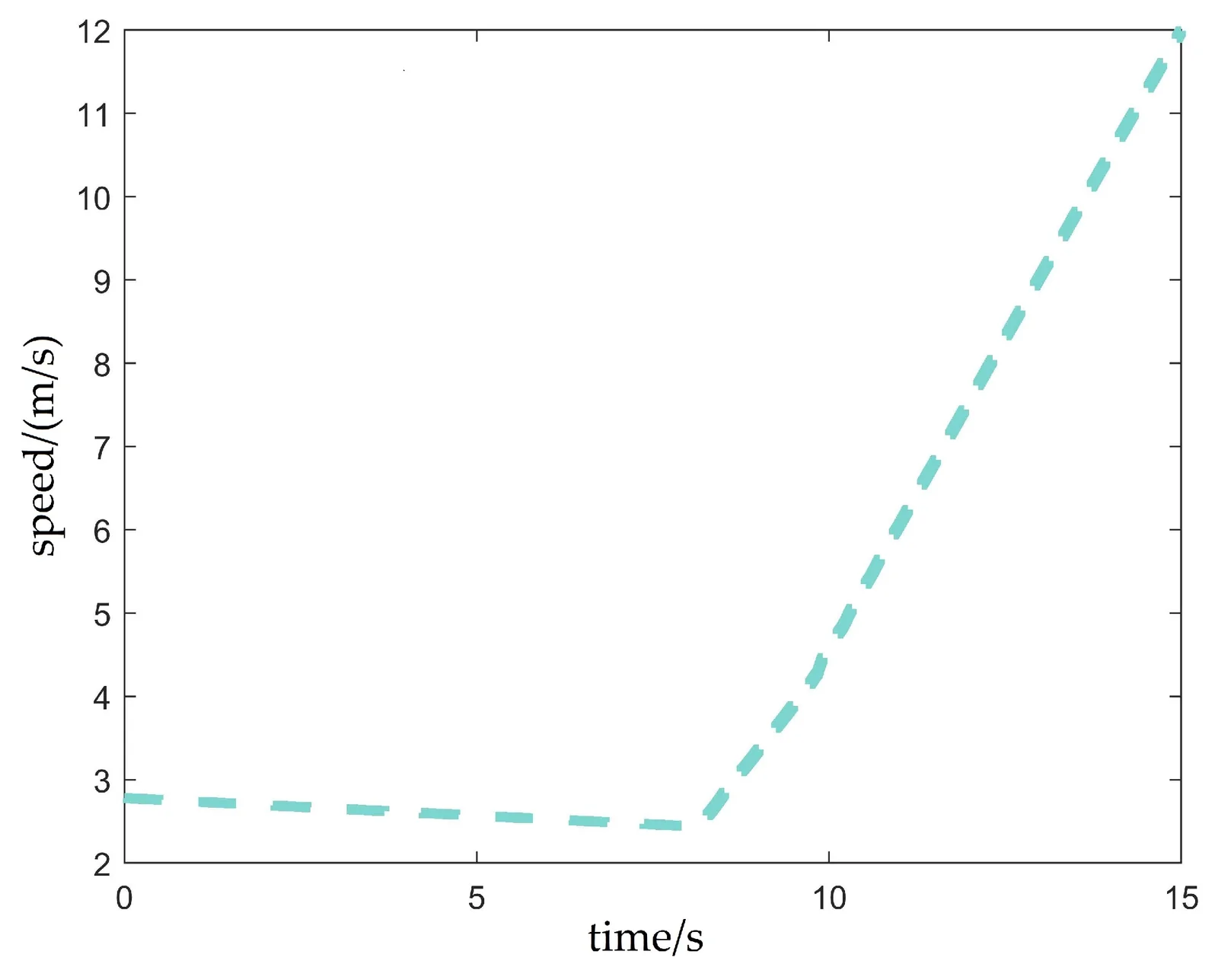
Downhill speed curve without control.

When the vehicle moves from a level road to a downhill slope, the force on the vehicle will change greatly, without additional control, the vehicle speed will increase sharply downhill., as shown in Figure 12.

**Figure 13:**
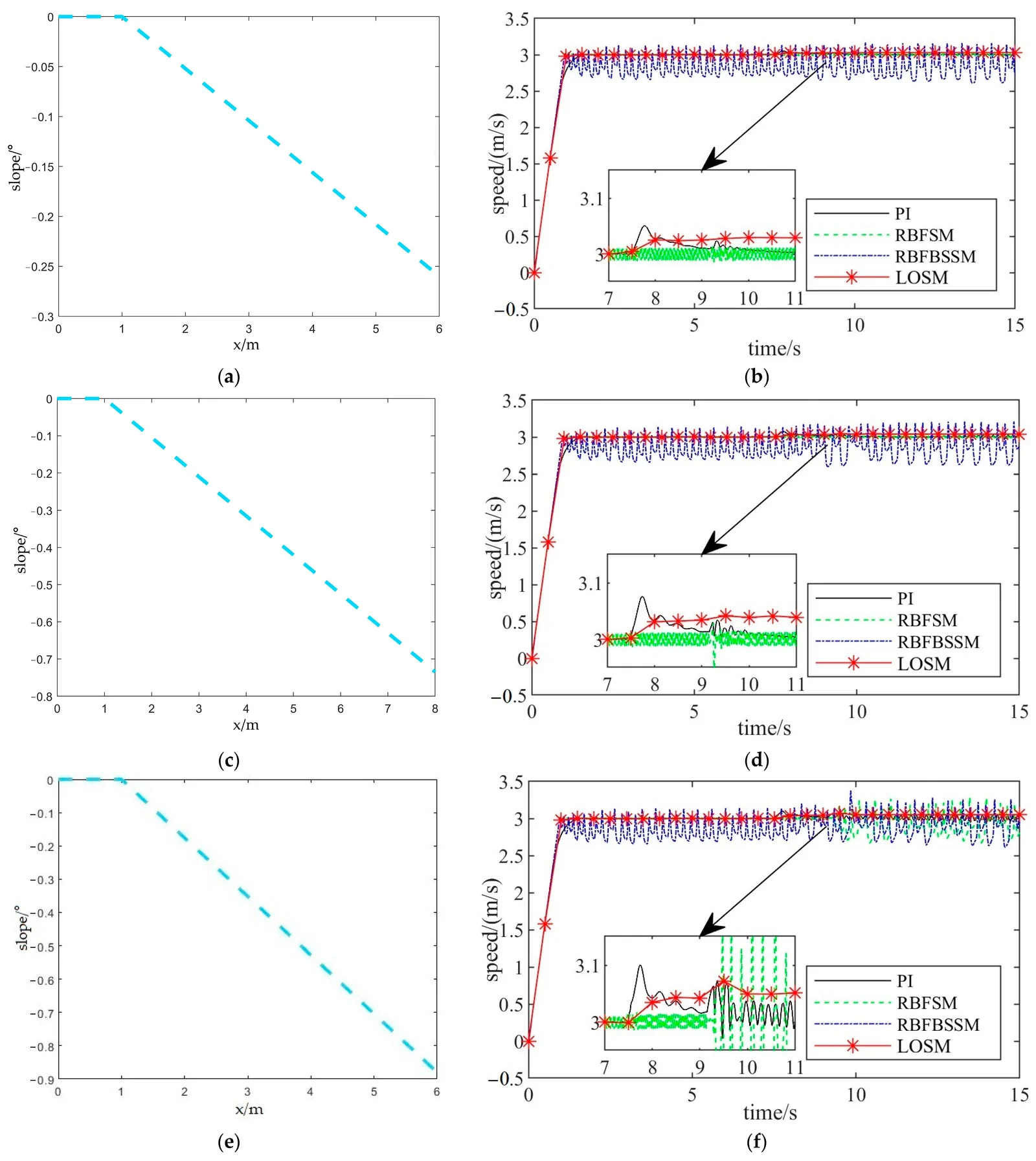
The control performance of certain load and gradient uncertain on downhill roads: (**a**) −3° slope; (**b**) speed under −3° slope; (**c**) −6° slope; (**d**) speed under −6° slope; (**e**) −10° slope; (**f**) speed under −10° slope.

**Figure 14:**
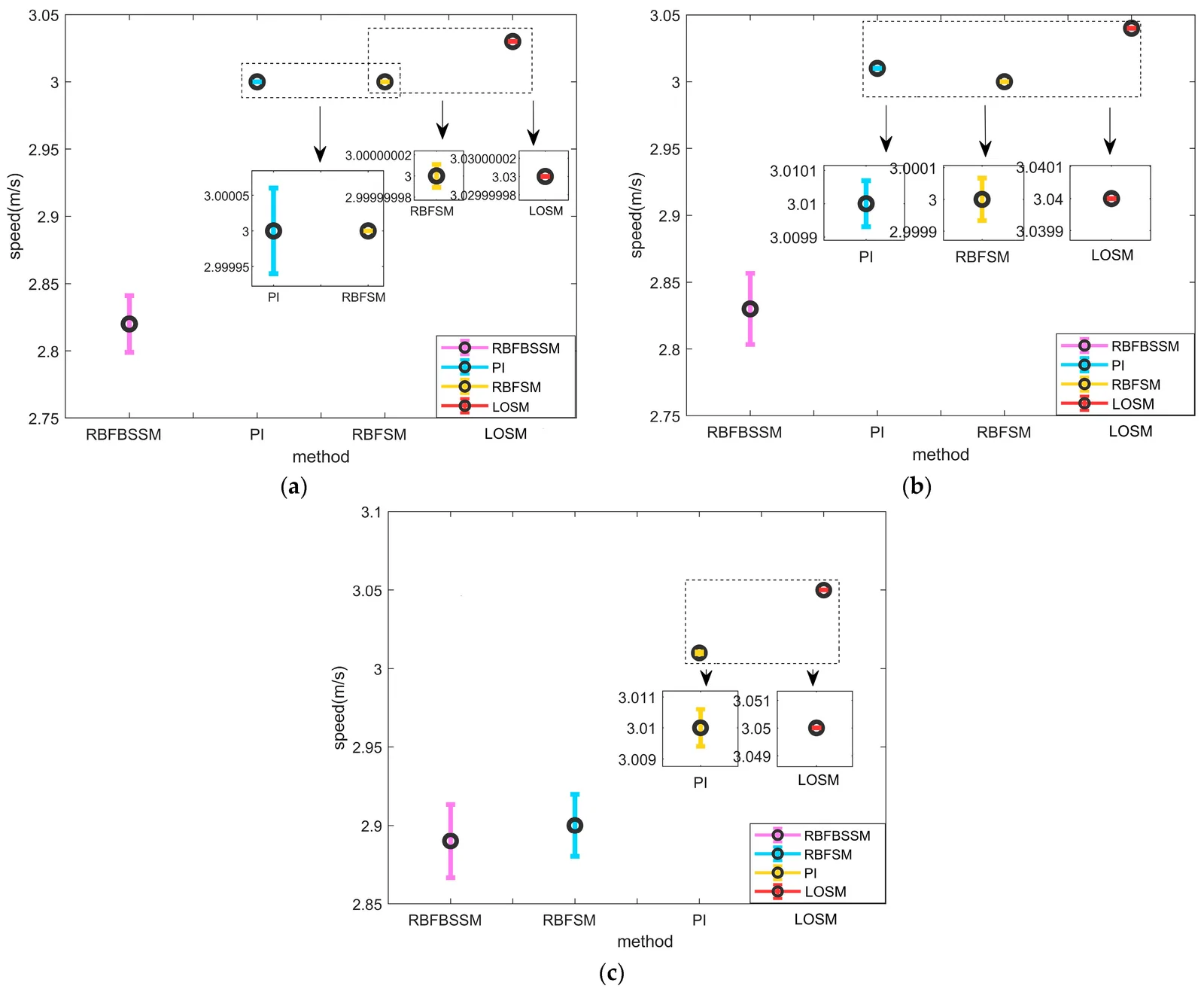
The speed error bars of certain load and gradient uncertain on downhill roads: (**a**) −3°; (**b**) −6°; (**c**) −10°.

For automated commercial vehicles, it is undesirable for the vehicle speed to deviate beyond a certain tolerance from the target speed. Instead, the speed is expected to be maintained at a stable value to facilitate systematic monitoring of safe vehicle operation. Consequently, an additional brake controller is required to regulate the vehicle speed. Moreover, given that commercial vehicles typically carry heavy payloads and thus possess significant inertia, excessive downhill speeds must be strictly avoided. In order to verify the effectiveness of LOSM in speed tracking in the face of downhill slope disturbance when the mass is certain, the slope is set to −3°, −6° and −10°, respectively, the target speed is set to 3 m/s, and the mass is set to 3000 kg. The control performances are shown in Figure 13 and Figure 14 and Table 5.

It can be observed from Figure 13 that, under different gradient conditions with identical vehicle mass, the LOSM controller is capable of maintaining a relatively stable vehicle speed. As shown in Table 5, the PI algorithm achieves the smallest steady-state mean error, followed by the RBFSM and LOSM algorithms, while the RBFBSSM algorithm exhibits the largest steady-state mean error. As illustrated in Figure 14, the error bars of the PI, RBFSM, and RBFBSSM algorithms are all significantly wider than those of the LOSM algorithm, indicating that the latter provides superior stability in speed control.

#### 4.2.6. Scenario 6: Load Uncertain and Gradient Uncertain in Downhill Road Driving

**Figure 15:**
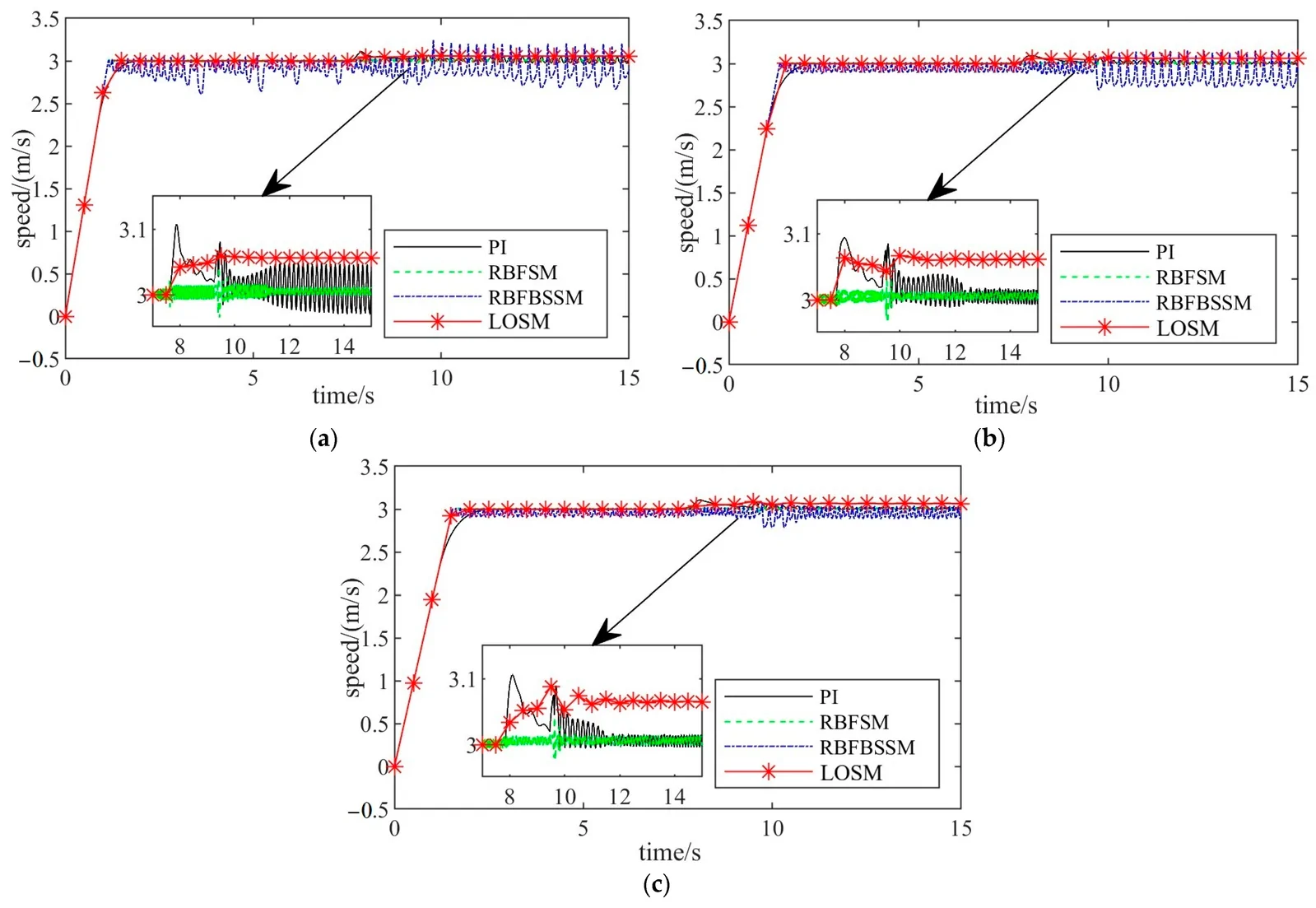
The control performance of uncertain load on gradient on downhill roads: (**a**) 4000 kg; (**b**) 5000 kg; (**c**) 6000 kg.

**Figure 16:**
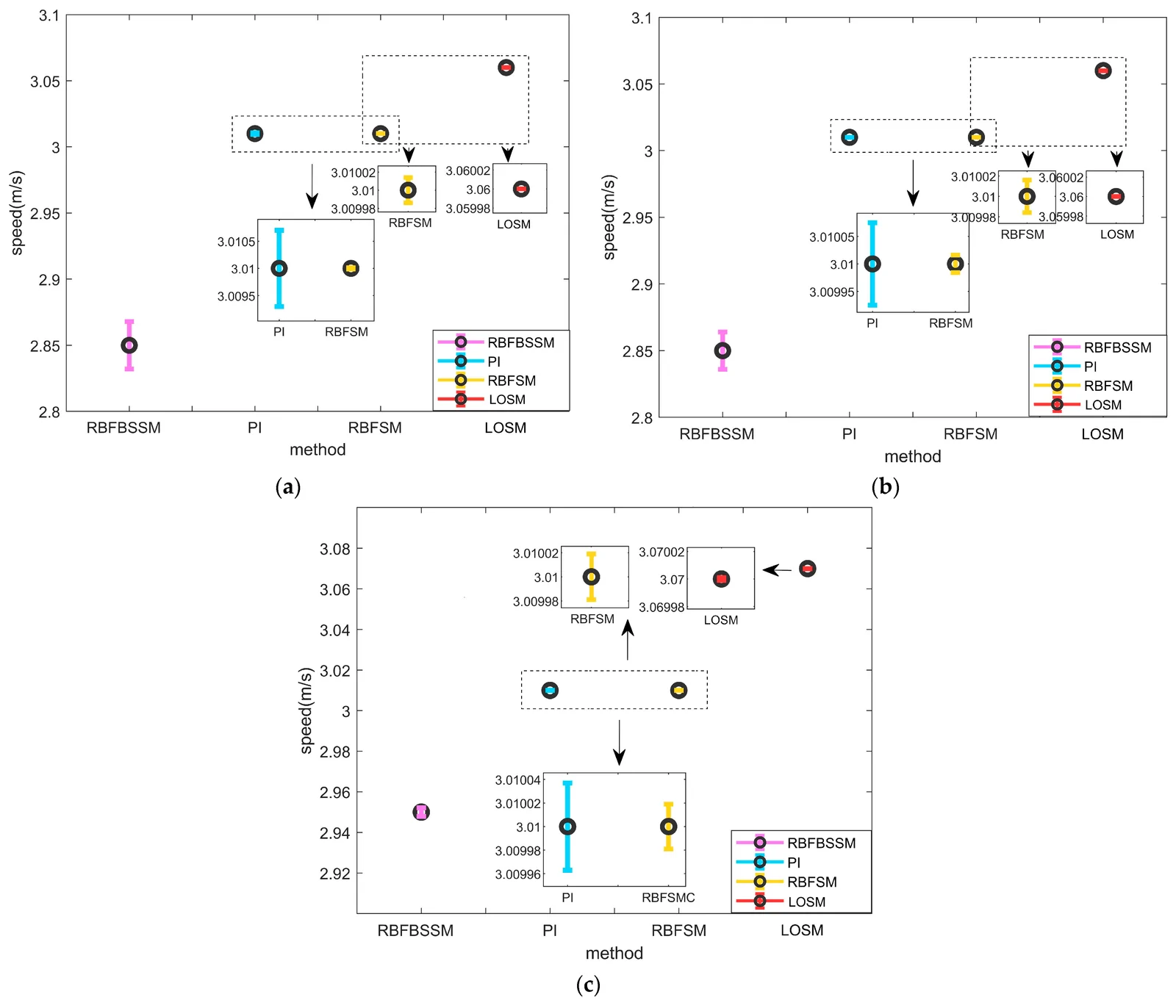
The error bar of uncertain load on gradient on downhill roads: (**a**) 4000 kg; (**b**) 5000 kg; (**c**) 6000 kg.

In order to verify the effectiveness of LOSM in speed tracking in the face of dual interference of load change and downhill slope change, compare the speed tracking performance on downhill roads. The vehicle mass is set to 4000 kg, 5000 kg and 6000 kg, respectively, and the slope is set to 10°, the target speed is set to 3 m/s. The control performances are shown in Figure 15 and Figure 16 and Table 6.

It can be observed from Figure 15 that, during downhill driving, the LOSM controller maintains relatively stable vehicle speeds for vehicles with different payload masses. In contrast, although the PI and RBFSM algorithms enable the vehicle speed to more closely approach the target value, they are accompanied by larger speed fluctuations. As shown in Table 6, the steady-state mean errors of the PI and RBFSM algorithms are smaller than that of the LOSM algorithm. However, as illustrated in Figure 16, the LOSM algorithm has the smallest error bar among all four algorithms, indicating its optimal stability in speed control. In other words, although the LOSM algorithm exhibits a larger steady-state mean error, its overall stability outperforms the other three algorithms.

#### 4.2.7. Scenario 7: Load Certain and Slope Uncertain in Comprehensive Road Driving

**Figure 17:**
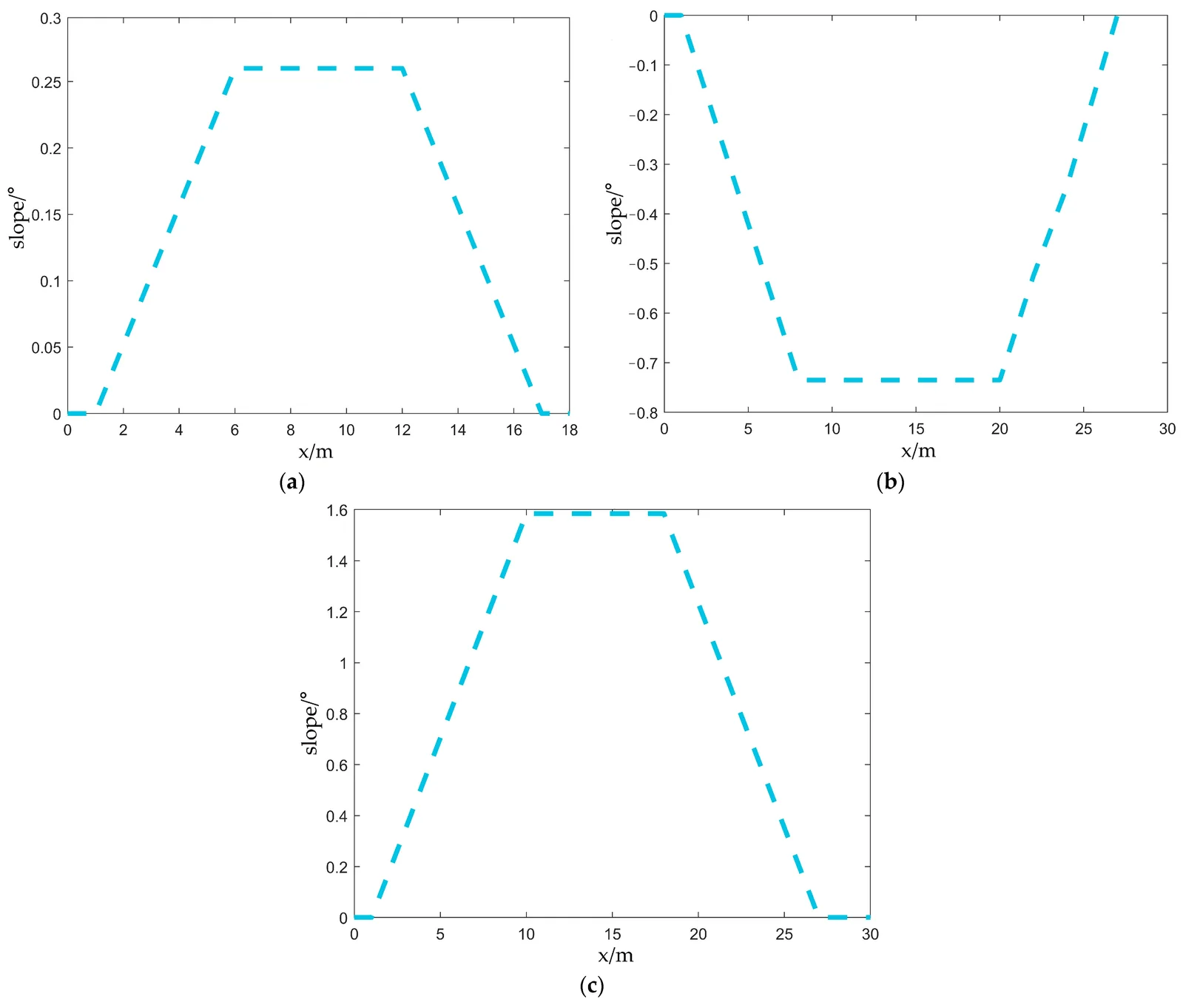
The comprehensive roads: (**a**) 3-(−3)° slope; (**b**) (−6)-6° slope; (**c**) 10-(−10)° slope.

**Figure 18:**
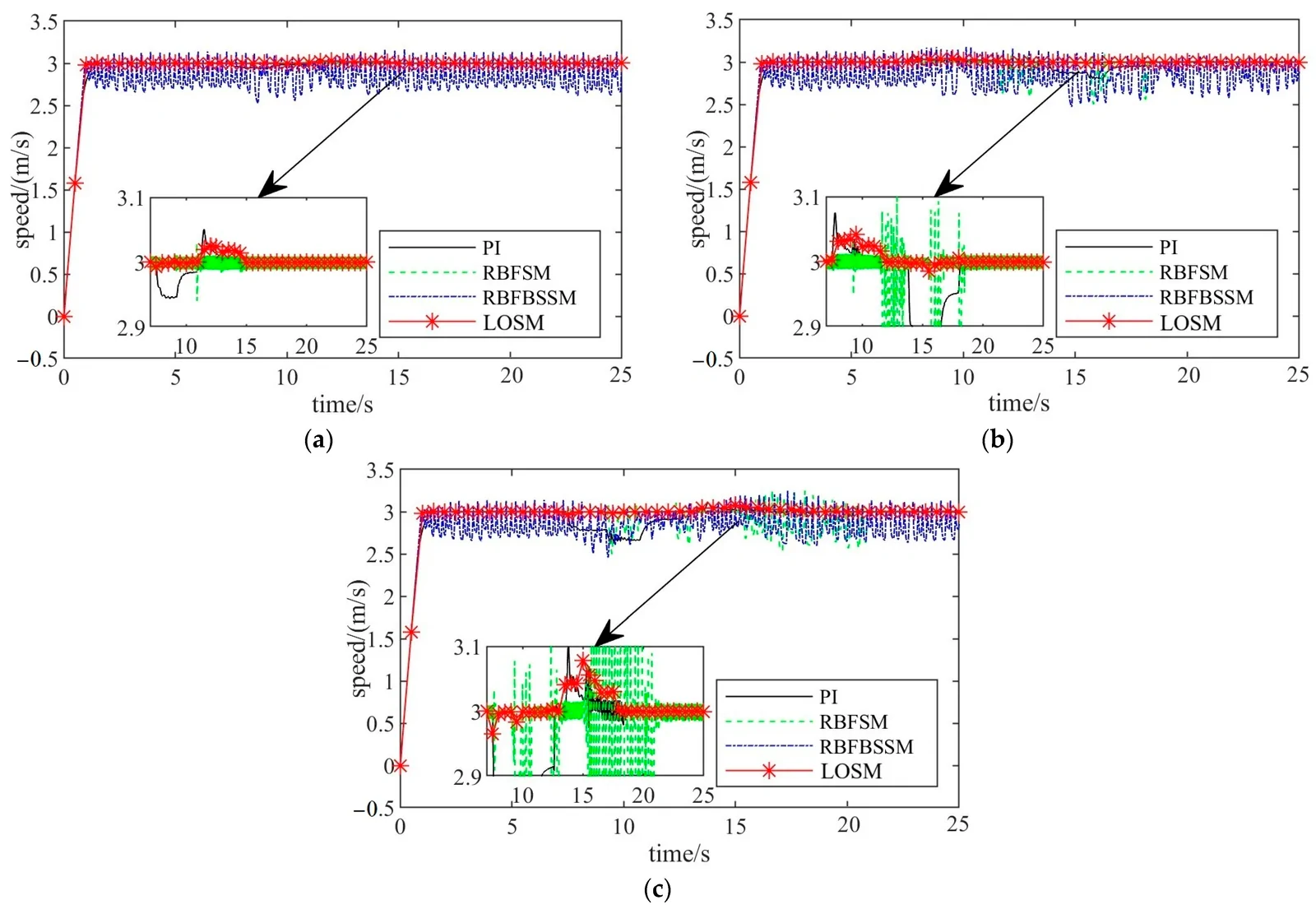
The control performance of certain load and slope uncertain on comprehensive roads: (**a**) 3-(−3)° slope; (**b**) (−6)-6° slope; (**c**) 10-(−10)° slope.

**Figure 19:**
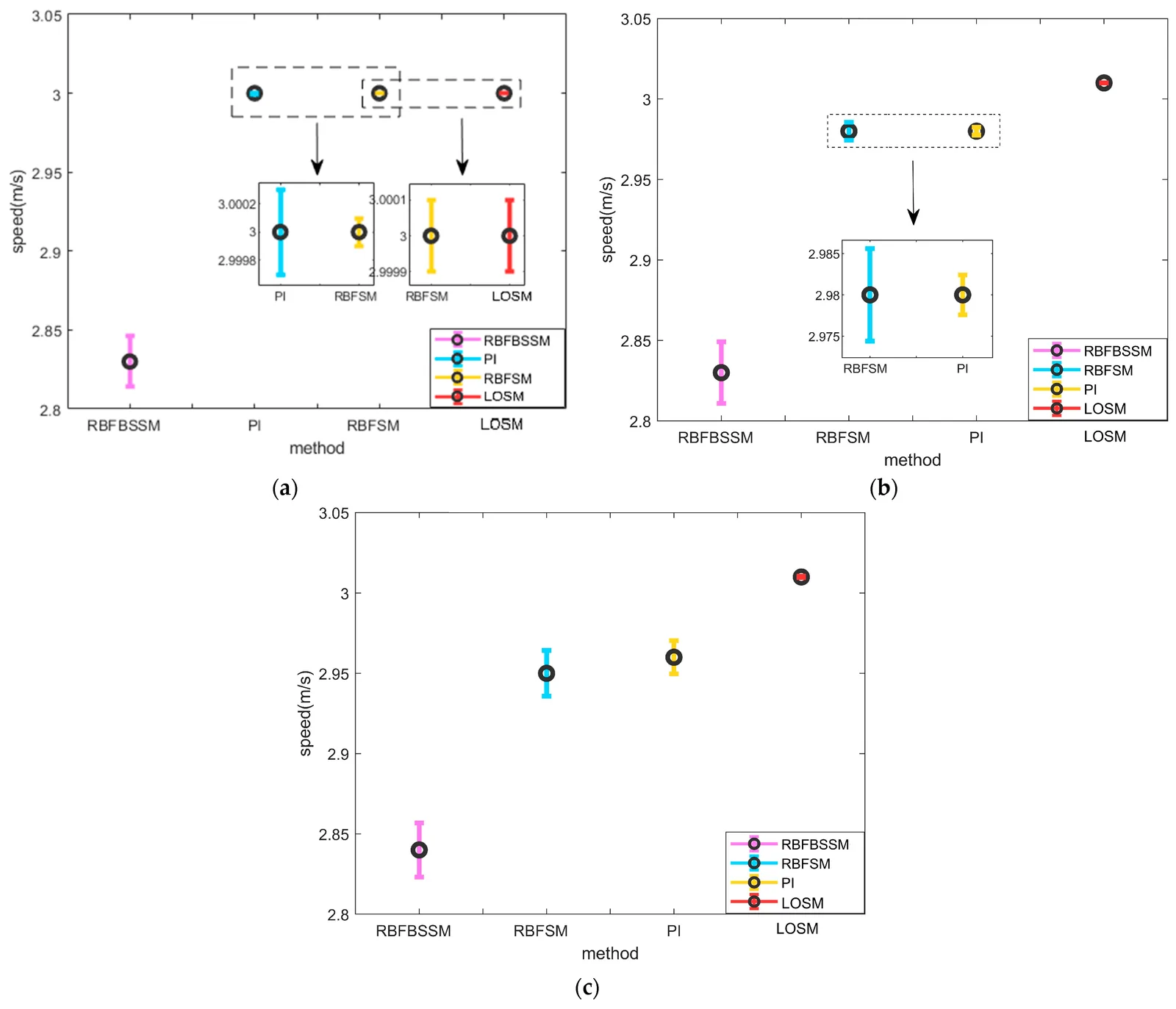
The error bar of certain load and slope uncertain on comprehensive roads: (**a**) 3-(−3)°slope; (**b**) (−6)-6° slope; (**c**) 10-(−10)° slope.

The above six scenarios have respectively completed the simulation tests for three single road types, flat road, uphill road, and downhill road. In order to verify the effectiveness of LOSM in speed tracking in the face of comprehensive slope interference when the load is certain, the road including level road, uphill road and downhill road scenes, the comprehensive road slope set to 3°, 6° and 10°, respectively, and the mass is set to 3000 kg. The comprehensive road definition is shown in Figure 17. The control performances are shown in Figure 18 and Figure 19 and Table 7.

Figure 18 shows that, under LOSM control, the vehicle can run smoothly at the desired speed under comprehensive road conditions and adapt quickly to road variations, whereas the comparison algorithms exhibit larger speed fluctuations and lower tracking accuracy. Table 7 indicates that LOSM achieves the smallest steady-state mean error. Figure 19 further reveals that LOSM has a narrower error bar than the other three algorithms. The results demonstrate that, whether the road changes abruptly from downhill to flat or from uphill to flat, the LOSM algorithm can effectively suppress the speed tracking error induced by gradient variations.

#### 4.2.8. Scenario 8: Load Uncertain and Slope Uncertain in Comprehensive Road Driving

**Figure 20:**
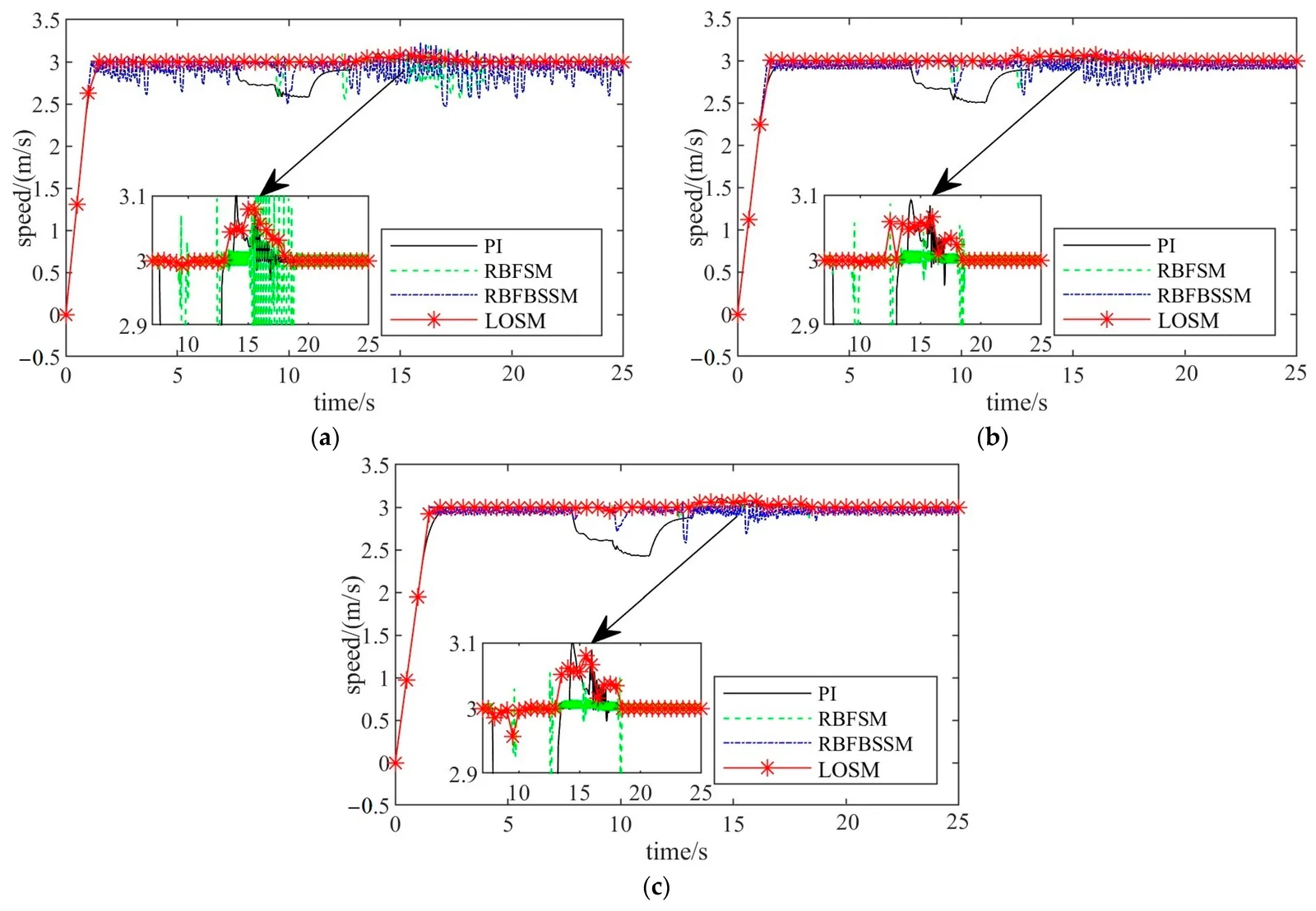
The control performance of uncertain load and slope uncertain on comprehensive roads: (**a**) 4000 kg; (**b**) 5000 kg; (**c**) 6000 kg.

**Figure 21:**
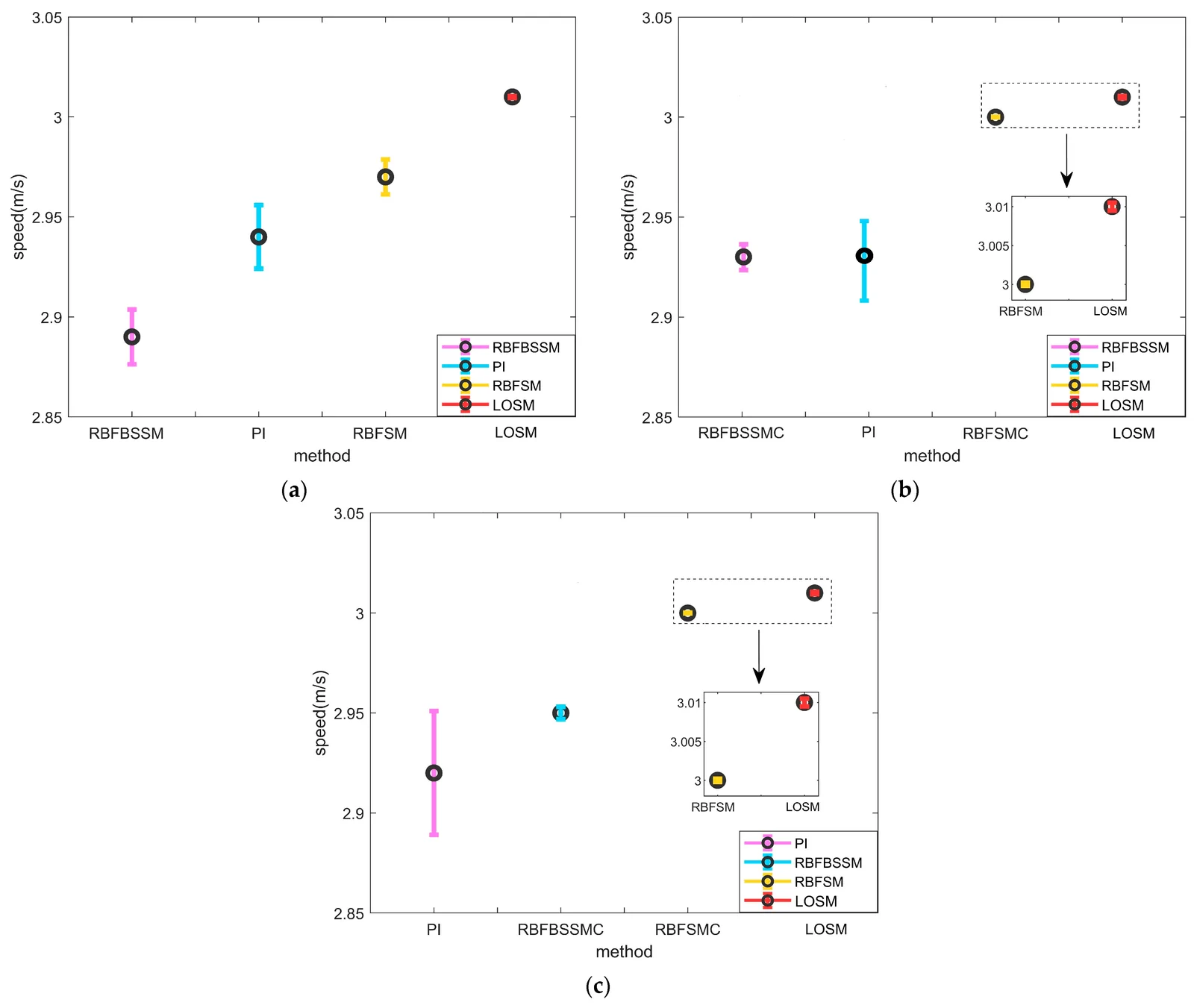
The error bar of uncertain load and slope uncertain on comprehensive roads: (**a**) 4000 kg; (**b**) 5000 kg; (**c**) 6000 kg.

The above experiments only carried out simulation tests on roads with different gradients. In order to verify the effectiveness of LOSM in speed tracking when faced with the dual interference of load change and comprehensive slope, the vehicle mass is set to 4000 kg, 5000 kg and 6000 kg, respectively, the road including level road, uphill road and downhill road scenarios, the comprehensive road slope is set to 10°, and the target speed is set at 3 m/s. The control performances are shown in Figure 20 and Figure 21 and Table 8.

As shown in Figure 20, under the dual uncertainties of payload and gradient, the LOSM controller enables the vehicle to travel at a relatively stable speed under comprehensive road conditions, while exhibiting rapid adaptability to road variations; in contrast, the comparison algorithms show more pronounced speed fluctuations. Table 8 indicates that the LOSM algorithm consistently maintains small steady-state mean errors under different payload conditions, demonstrating that the control performance is largely unaffected by payload variations. Figure 21 further reveals that the comprehensive error bars of LOSM under different payloads are narrower than those of the other three algorithms. The above results demonstrate that the LOSM algorithm can effectively suppress the adverse effects of abrupt gradient changes and mass variations on speed tracking performance.

### 4.3. Summary and Discussion of All Scenarios

A remark on tracking accuracy. For speed tracking in commercial vehicle applications, a steady-state error within ±5% is generally considered acceptable from an engineering perspective, while ±1% indicates a good level of performance. It should be noted that the extremely small error magnitudes observed in some cases are attributed to the idealized simulation environment adopted in this study, where sensor noise, actuator dynamics, and quantization errors are not modeled. These results demonstrate the theoretical effectiveness of the proposed observer–controller framework under nominal conditions. In real-world implementations, however, additional uncertainties would inevitably be introduced, which may degrade the tracking accuracy to some extent.

To provide a comprehensive overview of the controller performance across all designed scenarios, Table 9 summarizes the steady-state mean tracking errors for all eight scenarios, covering load uncertainty, gradient uncertainty, and their combinations. The results consistently demonstrate that the proposed LOSM strategy achieves the smallest or near-smallest tracking errors in the vast majority of cases, confirming its superior robustness against multi-source disturbances.

## 5. Conclusions

To address the uncertain disturbances of payload and road slope encountered by autonomous commercial vehicles (ACVs) during actual operations, this paper proposes a sliding mode control (SMC) strategy based on Luenberger observer disturbance compensation (LOSM). In the design of this strategy, a full-condition longitudinal dynamic model of the vehicle is first established. Subsequently, the Luenberger observer is deeply integrated with the SMC, and the boundary conditions for the asymptotic stability of the closed-loop system are rigorously derived using the Lyapunov stability theorem. To comprehensively verify the effectiveness of the proposed LOSM algorithm, eight typical testing scenarios are constructed, and three control algorithms are introduced for comparative analysis. The simulation results demonstrate that, across all testing scenarios, all four control algorithms can suppress the unknown mass and slope disturbances to a certain extent and accomplish the speed tracking task. Among them, the proposed LOSM strategy achieves the best overall performance in the majority of scenarios, demonstrating superior robustness under various combinations of load and gradient uncertainties. Although PI and RBFSM yield slightly smaller steady-state mean errors than LOSM in some downhill cases, the LOSM method achieves smaller error bars and smoother control responses in most operating conditions. These results demonstrate that the proposed method can significantly enhance the comprehensive speed control performance of ACVs in the presence of unknown payload and complex slope disturbances.

Future research will focus on incorporating multidimensional uncertainty factors, including but not limited to variations in vehicle geometric parameters and aerodynamic drag. In addition, since the current stability analysis is based on the constant-speed assumption, future work will extend the observer design and stability analysis to time-varying speed conditions. Moreover, investigating the impact of multi-source parameter uncertainties on the vehicle’s lateral control performance holds significant research value. Related robust adaptive control strategies developed for underactuated systems, such as the low-frequency learning-gain approach [28], may provide useful insights for addressing similar challenges in vehicle lateral dynamics. Finally, continuously conducting real-vehicle testing and iteratively optimizing the control strategy based on feedback from real-world physical environments will be essential steps in driving this technology toward practical engineering deployment.

## Figures and Tables

**Table 1:** The steady-state mean error of certain load on level roads.

Mass (kg)	Speed (m/s)	Slope (°)	Steady-State Mean Error (m/s)			
			PI	RBFSM	RBFBSSM	LOSM
3000	3	0	0	0	0.19	0
3000	10	0	0	0.28	0.31	0

**Table 2:** The steady-state mean error of uncertain load on level roads.

Mass (kg)	Slope (°)	Steady-State Mean Error (m/s)			
		PI	RBFSM	RBFBSSM	LOSM
4000	0	0	0	0.09	0
5000	0	0	0	0.06	0
6000	0	0	0	0.05	0

**Table 3:** The steady-state mean error of certain load and gradient uncertain on uphill roads.

Mass (kg)	Slope (°)	Steady-State Mean Error (m/s)			
		PI	RBFSM	RBFBSSM	LOSM
3000	3	0.08	0	0.2	0
3000	6	0.19	0.18	0.06	0
3000	10	0.33	0	0.11	0

**Table 4:** The steady-state mean error of uncertain load on gradient on uphill roads.

Mass (kg)	Slope (°)	Steady-State Mean Error (m/s)			
		PI	RBFSM	RBFBSSM	LOSM
4000	10	0.4	0	0.01	0
5000	10	0.47	0.01	0.01	0.01
6000	10	0.55	0.01	0.01	0.01

**Table 5:** The steady-state mean error of certain load and gradient uncertain on downhill roads.

Mass (kg)	Slope (°)	Steady-State Mean Error (m/s)			
		PI	RBFSM	RBFBSSM	LOSM
3000	−3	0	0	0.18	0.03
3000	−6	0.01	0	0.17	0.04
3000	−10	0.01	0.01	0.11	0.05

**Table 6:** The steady-state mean error of uncertain load on gradient on downhill roads.

Mass (kg)	Slope (°)	Steady-State Mean Error (m/s)			
		PI	RBFSM	RBFBSSM	LOSM
4000	−10	0.01	0.01	0.15	0.06
5000	−10	0.01	0.01	0.15	0.06
6000	−10	0.01	0.01	0.05	0.07

**Table 7:** The steady-state mean error of load certain and slope uncertain on comprehensive roads.

Mass (kg)	Slope (°)	Steady-State Mean Error (m/s)			
		PI	RBFSM	RBFBSSM	LOSM
3000	3-(−3)	0	0	0.17	0
3000	(−6)-6	0.02	0.02	0.17	0.01
3000	10-(−10)	0.04	0.05	0.16	0.01

**Table 8:** The steady-state mean error of uncertain load and slope uncertain on comprehensive roads.

Mass (kg)	Slope (°)	Steady-State Mean Error (m/s)			
		PI	RBFSM	RBFBSSM	LOSM
4000	10-(−10)	0.06	0.03	0.11	0.01
5000	10-(−10)	0.07	0	0.07	0.01
6000	10-(−10)	0.08	0	0.05	0.01

**Table 9:** Summary of steady-state mean tracking errors for all eight scenarios.

Scenario	Description	Steady-State Mean Error (m/s)			
		PI	RBFSM	RBFBSSM	LOSM
1	Certain load, level road (3/10 m/s)	0/0/0	0/0.28	0.19/0.31	0/0/0
2	Uncertain load, level road (4000/5000/6000 kg)	0/0/0	0/0/0	0.09/0.06/0.05	0/0/0
3	Certain load, uphill with gradient uncertainty (3°/6°/10°)	0.08/0.19/ 0.33	0/0.18/0	0.2/0.06/0.11	0/0/0
4	Uncertain load, uphill with gradient uncertainty (4000/5000/6000 kg)	0.4/0.47/0.55	0/0.01/0.01	0.01/0.01/0.01	0/0.01/0.01
5	Certain load, downhill with gradient uncertainty (−3°/−6°/−10°)	0/0.01/0.01	0/0/0.01	0.18/0.17/0.11	0.03/0.04/0.05
6	Uncertain load, downhill with gradient uncertainty (4000/5000/6000 kg)	0.01/0.01/ 0.01	0.01/0.01/ 0.01	0.15/0.15/0.05	0.06/0.06/0.07
7	Certain load, comprehensive road with slope uncertainty (3-(−3)°/(−6)-6°/10-(−10)°)	0/0.02/0.04	0/0.02/0.05	0.17/0.17/0.16	0/0.01/0.01
8	Uncertain load, comprehensive road with slope uncertainty (4000/5000/6000 kg)	0.06/0.07/ 0.08	0.03/0/0	0.11/0.07/0.05	0.01/0.01/0.01

## Data Availability

The data presented in this study are available on request from the corresponding author due to privacy.
